# Experiences of coordinated care for people in the UK affected by rare diseases: cross-sectional survey of patients, carers, and healthcare professionals

**DOI:** 10.1186/s13023-023-02934-9

**Published:** 2023-11-23

**Authors:** Holly Walton, Pei Li Ng, Amy Simpson, Lara Bloom, Lyn S. Chitty, Naomi J. Fulop, Amy Hunter, Jennifer Jones, Joe Kai, Larissa Kerecuk, Maria Kokocinska, Kerry Leeson-Beevers, Sharon Parkes, Angus I. G. Ramsay, Alastair Sutcliffe, Christine Taylor, Stephen Morris

**Affiliations:** 1 Department of Applied Health Research, University College London, Gower Street, London, WC1E 6BT UK; 2 Genetic Alliance UK, Creative Works, 7 Blackhorse Lane, London, E17 6DS UK; 3 The Ehlers-Danlos Society and Academic Affiliate Professor of Practice in Patient Engagement and Global Collaboration (Penn State College of Medicine), Hershey, USA; 4 North Thames Genomic Laboratory Hub, Great Ormond Street NHS Foundation Trust, London, UK; 5 UCL Great Ormond Street Institute of Child Health, London, UK; 6 Division of Primary Care, Centre for Academic Primary Care, NIHR School for Primary Care Research, University of Nottingham, Floors 13-15, Tower Building, University Park, Nottingham, NG7 2RD UK; 7 Birmingham Women’s and Children’s NHS Foundation Trust, Birmingham, UK; 8 NIHR Clinical Research Network West Midlands, Birmingham, UK; 9 Alstrom Syndrome UK, 4 St Kitts Close, Torquay, TQ2 7GD Devon UK; 10 UCL and Great Ormond Street Institute of Child Health, 30 Guilford Street, London, WC1N 1EH UK; 11 Primary Care Unit, Department of Public Health and Primary Care, University of Cambridge, Cambridge, UK

**Keywords:** Rare conditions, Rare diseases, Care coordination, Survey, Care plan, Care coordinator, Specialist centre

## Abstract

**Background:**

Poorly coordinated care can have major impacts on patients and families affected by rare conditions, with negative physical health, psychosocial and financial consequences. This study aimed to understand how care is coordinated for rare diseases in the United Kingdom.

**Methods:**

We undertook a national survey in the UK involving 760 adults affected by rare diseases, 446 parents/carers of people affected by rare diseases, and 251 healthcare professionals who care for people affected by rare diseases.

**Results:**

Findings suggested that a wide range of patients, parents and carers do not have coordinated care. For example, few participants reported having a care coordinator (12% patients, 14% parents/carers), attending a specialist centre (32% patients, 33% parents/carers) or having a care plan (10% patients, 44% parents/carers). A very small number of patients (2%) and parents/carers (5%) had access to all three—a care coordinator, specialist centre and care plan. Fifty four percent of patients and 33% of parents/carers reported access to none of these. On the other hand, a higher proportion of healthcare professionals reported that families with rare conditions had access to care coordinators (35%), specialist centres (60%) and care plans (40%).

**Conclusions:**

Care for families with rare conditions is generally not well coordinated in the UK, with findings indicating limited access to care coordinators, specialist centres and care plans. Better understanding of these issues can inform how care coordination might be improved and embrace the needs and preferences of patients and families affected by rare conditions.

**Supplementary Information:**

The online version contains supplementary material available at 10.1186/s13023-023-02934-9.

## Background

Rare conditions affect over 3.5 million people in the United Kingdom [1] and more than 300 million worldwide [2]. Although each rare condition affects fewer than 1 in 2000 people [3], there are over 6000 rare conditions in total [2]. Rare conditions affect both children and adults, and are often lifelong, chronic and complex in nature. Rare conditions often affect multiple systems of the body, requiring mental and physical health support and requiring patients to see many different specialists [1].

To ensure that families living with rare conditions receive the support they need, care may need to be coordinated. An updated definition of care coordination, in the context of rare conditions, highlights that for care to be coordinated, everyone involved in a patient’s care (including the patient and carer) should work together across multiple components and processes of care in order to achieve shared outcomes [4]. The definition highlighted that care should be coordinated throughout a person’s whole life, across all parts of the healthcare system, be family-centred, holistic, evidence-based and accessible for all patients with rare conditions irrespective of the condition, situation or location [4]. Examples of components of care coordination include patients attending multidisciplinary or joint clinics, having care coordinators, and having shared electronic records [4].

However, the complex nature of the care pathway for patients and families with rare diseases means that often the care they receive is not coordinated, and is in fact disjointed. For example, families may have to travel long distances to visit lots of different specialists, care plans may not be in place or followed, and professionals may not always share information between themselves, putting the care burden onto families [5]. To potentially reduce this burden of care, it is important to ensure that care is coordinated for patients and families living with rare conditions.

Despite the need to coordinate care, previous research has highlighted that individuals living with rare conditions experience a lack of care coordination; resulting in often disjointed care [4–7]. For example, findings from a survey of patients and families affected by rare diseases indicated problems with services not sharing test results and information between services, problems with patients travelling a long way to attend multiple appointments, lack of care coordinators and lack of access to specialist centres [5]. Additionally, findings from a qualitative exploratory study on the impacts of coordinated care highlighted that a lack of care coordination can have a negative impact on patients’ and families’ psychological and social situation (e.g. emotional burden, attendance at school), physical health, and financial situation (e.g. travel costs) [6].

Recently, there have been a range of policy drivers introduced throughout the United Kingdom (UK) that outline care coordination as a key priority and indicate the need to strive towards improving coordinated care for those living with rare conditions [8–13].

While previous research indicated the importance of care coordination for rare conditions, to the authors’ knowledge, little research has focused on exploring what levels of care coordination patients and carers receive within the UK. This study therefore aimed to fill this gap by exploring what levels of care coordination patients, carers and healthcare professionals report across the UK.

The study aimed to explore how care is coordinated in the UK for people affected by rare conditions and addressed the following research questions:

1. What levels of access to combinations and individual elements of care coordination (care coordinators, specialist centres, care plans) do patients and carers report?
2. What levels of access to elements of care coordination (care coordinators, specialist centres, care plans) do healthcare professionals report?

## Methods

### Design

A cross-sectional large national (UK) survey of patients, parents and carers, and healthcare professionals was conducted.

### Ethical approval

The CONCORD study received ethical approval from UCL Research Ethics Committee (8423/002) and the London-Surrey Borders Research Ethics Committee of the Health Research Authority (19/LO/0250).

### Survey instrument

#### Development of survey

We developed the survey specifically for this study. The survey was developed using data from three sources. First, we identified themes from a scoping review of 154 reviews (including narrative reviews, meta-analyses, systematic reviews and scoping reviews) of coordinated care for rare and chronic conditions [4] to identify important components of coordinated care. Second, we ran three focus groups involving patients aged over 18 years affected by a rare condition, parents/carers of children and adults affected by a rare condition, and healthcare professionals involved in the treatment of rare conditions. One focus group was conducted virtually with four patients and three carers (recruited through charity partners); two were conducted face-to-face, one with four healthcare professionals, the other with two patients and four parents/carers [4]. Third, we ran 15 one-to-one telephone or Skype interviews involving seven patients and eight parents/carers (recruited through charity partners) [6]. Using the findings from these activities we identified three key areas of care coordination that mattered to patients and families: (1) access to care coordinators; (2) being able to attend specialist centres; and (3) having care plans. Within our survey, we outlined definitions of these areas of care coordination (see Table 1).

**Table 1:** Key definitions of care coordination areas included in survey

Area of care coordination	Definition
Care coordinator	‘A professional with a recognised role in helping patients and carers manage a range of needs between different professionals or across care settings. They may be a full-time coordinator or may coordinate care as part of their main job, such as a GP’
Specialist centre	‘A centralised facility that enables patients to see a number of healthcare professionals in one visit. Usually, the professionals at specialist centres will be experts in rare and undiagnosed conditions. Non-healthcare professionals may also see patients at the same centre’
Care plan	'A paper or electronic document which describes the health services and support that are needed and should be agreed between patients, carers and professionals. The care plan may be a single document or it may be part of another record which includes non-health services such as an Education, Health and Care Plan (EHCP)’

We developed a first draft of the questionnaire to understand how care of people with rare conditions was coordinated in the UK specifically with reference to these items.

The survey included: (1) a section on consent and participant eligibility (including a participant information sheet), (2) experiences of diagnosis and rare conditions, (3) experience of care coordinators, (4) experience of specialist centres, (5) experience of care plans, (6) use of health services, and (7) socio-demographic factors (see Additional files 1, 2 and 3: Appendix S1–S3). Most questions were closed questions with defined responses. A minority of questions were open questions, to enable participants to provide free-text responses (e.g. the rare condition they were affected by).

The draft survey was reviewed by the CONCORD Patient and Public Involvement Advisory Group (PPIAG). The group provided feedback on the language and content of the survey.

#### Piloting of survey

The questionnaire was then piloted in two ways: (1) Twenty respondents completed the questionnaire on their own and provided feedback in writing, (2) we conducted four ‘think-aloud’ interviews [14] with four participants (one patient, two parents and a healthcare professional). Think aloud interviews are structured interviews, in which the participant completed the questionnaire in the presence of a researcher, and the participants are asked to talk through their thought processes [14].

We then amended the survey according to feedback and the PPIAG reviewed the amended survey to ensure that the language used was comprehensible and relevant to the intended focus of each question.

### Survey sampling

Participants were all from the UK. Three groups of participants were eligible to complete the survey: (a) patients (aged over 18 years) affected by a rare condition; (b) parents and carers (aged ≥ 18 years) of children or adults with rare conditions; and, (c) healthcare professionals (doctors, nurses and allied health professionals) involved in the care of people with rare conditions.

We aimed to recruit at least 300 participants for each group, with an overall target sample size of 1500. The 1500 figure was justified using two pieces of information. First, sample size calculations for surveys are possible based on population size, desired confidence level and maximum acceptable margin of error. Assuming a population size of upwards of 20,000 (predicted sample size remains close to constant for populations larger than 20,000), a margin of error of 3%, and a confidence level of 95%, the required sample size is 1014 [15]. Second, our target figure of 1500 was partly informed by another UK survey on rare diseases which used a similar research design and achieved a sample size of 1213 [5].

There were no restrictions on participants in terms of the rare condition, demographic factors (other than age ≥ 18 years), or geographical location within the UK. We deliberately did not sample from specific rare diseases, nor limit the range of rare diseases we included, to include as broad a range of experiences with regards to care coordination as possible. A complete sample frame of all adults living with a rare condition in the UK does not exist: the total number of people living with a rare condition, their contact details and their socio-demographic characteristics such as age, gender, highest education level and location of residence are unknown. For these reasons, convenience snowball sampling was used for this study. We discussed routes to accessing patients and parents/carers with the PPIAG. Participants were accessed via patient and provider networks and organisations, including Rare Disease UK (which has more than 2000 registered supporters including academics, clinicians, industry, individual members and patient organisations [16]); Genetic Alliance UK (a national alliance of organisations with a membership of more than 180 charities supporting patients and families affected by genetic disorders [17]; SWAN UK (Syndromes Without A Name; a support network for families of children and young adults with undiagnosed genetic conditions in the UK run by Genetic Alliance UK [18]) and Breaking Down Barriers [19]. We relied on self-reported diagnoses of rare conditions and did not verify diagnoses.

### Procedure

An independent survey company created an electronic version of the survey using a bespoke online platform. The survey was ‘live’ from August to December 2019. Potential participants were sent a weblink to the survey either by email or social media. The message containing the weblink also included an offer to send hard copies of the questionnaire by post or email or to complete it verbally over the telephone with a researcher. We also recruited patients and parents/carers via six major care providers, where research coordinators at each site identified potential participants and asked if they were willing to participate. If they were willing to participate, they were provided with further details on how to do this, as described above. Healthcare professionals were recruited using the same routes described above for patients and parents/carers. In addition, we contacted the British Society for Genetic Medicine and its constituent organisations and special interest groups [20], and the NIHR Clinical Research Network: Genetics [21]. These organisations circulated details of the survey to their members via their email lists. Participants had a 48-h window where they were able to suspend completion of the questionnaire, if they needed to do so, and then to resume where they left off at a time that was convenient to them.

Taking part was voluntary, participants gave implied consent at the time of starting the survey having received study information.

### Analysis of data

All data handling was conducted in compliance with General Data Protection Regulation requirements and all responders agreed to their data being processed for research purposes. Anonymised data were transferred from the survey company into a secure Data Safe Haven for analysis by the researchers via a secure File Transfer Protocol system. Responses where less than 20% of all data fields were completed were removed. All the quantitative results except for those relating to the use of health services were reported as frequencies and percentages using Microsoft Excel [22] and Stata [23]. For the use of services we reported the mean values per patient, stratified by whether or not the patient had access to a care coordinator, a specialist centre and a care plan. We did not impute missing data.

## Results

### Participant characteristics

#### Summary

We received 1604 survey responses, including from patients (n = 856), parents or carers (n = 497) and healthcare professionals (n = 251). Responses with less than 20% of data fields completed were excluded (n = 96, 11% patients; n = 51, 10% carers). This resulted in a final sample of 1457 responses (n = 760 patients, n = 446 parents/carers, n = 251 healthcare professionals). Due to multiple overlapping distribution methods (using convenience and snowball sampling), it was not possible to calculate a response rate.

#### Patients and carers

Patients and parents/carers were recruited from all regions in the UK. More participants were female (patients: n = 434, 85%; carers: n = 235, 88%) than male (patients: n = 73, 14%, carers: n = 32, 12%). Most participants reported their ethnicity as white. For patients, the most common age ranges of respondents were between 45–64 (45–54: n = 124, 24%; aged 55–64: n = 115, 23%) and for parents and carers, the most common age ranges of respondents were between 35–54 (35–44: n = 86, 32%, 45–54: n = 94, 35%).

More parents/carers reported caring for a child up to the age of 18 (n = 181; 68%), compared to those reporting caring for an adult over 18 (n = 87; 19%). The majority of parents/carers were the parent of a patient with a rare condition (n = 192, 71%), though a small percent were either the son or daughter (n = 41, 15%) or spouse or partner (n = 23, 9%) of a person living with a rare condition.

The majority of participants had received a rare disease diagnosis (patients: n = 736; 98%; carers: n = 400, 91%), but fewer participants had diagnoses confirmed by genetic testing (patients: n = 223, 30%; carers: n = 255, 64%). Collectively, patients reported 221 rare diseases, and carers reported 259 rare diseases. See Table 2 for more information.

**Table 2:** Sample characteristics of patients and parents/carers

	Characteristic	Patients (n = 760)			Parents/carers (n = 446)	
		Number	%		Number	%
Age of patient (years)	0–5	N/A	N/A		66	25
	6–12	N/A	N/A		81	30
	13–17	N/A	N/A		34	13
	18–24	21	4		33	12
	25–34	75	15		18	7
	35–44	94	18		8	3
	45–54	124	24		11	4
	55–64	115	23		12	4
	65–74	66	13		4	1
	≥ 75	14	3		1	0
	Total	509	100		268	100
	Prefer not to say	3			3	1
	Missing	248			175	39
Age of parent/carer (years)	18–24	N/A	N/A		5	2
	25–34	N/A	N/A		36	13
	35–44	N/A	N/A		94	35
	45–54	N/A	N/A		86	32
	55–64	N/A	N/A		36	13
	65–74	N/A	N/A		11	4
	≥ 75	N/A	N/A		1	0
	Total	N/A	N/A		269	100
	Prefer not to say	N/A	N/A		2	
	Missing	N/A	N/A		175	
Sex	Male	73	14		32	12
	Female	434	85		235	88
	Other	2	0		1	0
	Total	509	100		268	100
	Prefer not to say	3			3	
	Missing	248			175	
Diagnosed with rare disease	Yes	736	98		400	91
	No	17	2		38	8
	Unsure	7	1		8	2
	Total	760	100		446	100
Diagnosis confirmed with genetic test	Yes	223	30		255	64
	No	402	55		110	28
	Unsure	111	15		35	9
	Total	736	100		400	100
	Not applicable (undiagnosed)	24			46	
Body system affected	Muscle, ligaments, joints (rheumatology)	438	58		232	52
	Vision	432	57		114	26
	Brain, nerves, spinal cord (neurology)	345	45		229	51
	Digestion (gastroenterology)	337	44		222	50
	Hearing	327	43		201	45
	Bones, joints (orthopaedics)	318	42		205	46
	Skin (dermatology)	314	41		129	29
	Breathing, lungs (respiratory)	302	40		175	39
	Chronic pain	296	39		166	37
	Heart, circulatory (cardiology)	230	30		159	36
	Diabetes, hormones (endocrinology)	188	25		95	21
	Kidneys (nephrology)	178	23		115	26
	Behavioural difficulties	153	20		128	29
	Learning difficulties	65	9		175	39
	Mental health (psychiatry)	35	5		229	51
	Total	760			446	
Number of rare diseases in sample		221			259	
Top 10 most common rare diseases in sample (reported by patients, and reported by parents/carers)	1. Sarcoidosis	101	13	1. Behçet's syndrome	18	4
	2. Behçet's syndrome	85	11	2. Trachea oesophageal fistula	14	3
	3. Idiopathic intercranial hypertension	49	6	3. Aplastic anaemia	9	2
	4. Lynch syndrome	26	3	4. Ataxia	8	2
	5. Ehlers-Danlos syndrome	24	3	5. Rett syndrome	6	1
	6. IgA nephropathy	24	3	6. Tuberous sclerosis	6	1
	7. Ocular melanoma	17	2	7. Common variable immune deficiency	5	1
	8. Common variable immunedeficiency	14	2	8. Dravet syndrome	5	1
	9. Scleroderma	12	2	9. Huntington’s disease	5	1
	10. Allergic broncho pulmonary aspergillosis (ABPA)	11	1	10. Multiple system atrophy	5	1
Patient’s living arrangements	Lives alone	115	23		N/A	N/A
	Lives with a spouse or partner	289	57		N/A	N/A
	Lives with family members or friends	99	19		N/A	N/A
	Lives with a carer	2	1		N/A	N/A
	Total	505	100		N/A	N/A
	Prefer not to say	7			N/A	N/A
	Missing	248			N/A	N/A
Parent’s/carer’s relationship to patient	Spouse or partner	N/A	N/A		23	9
	Parent	N/A	N/A		192	71
	Guardian	N/A	N/A		3	1
	Grandparent	N/A	N/A		2	1
	Sibling	N/A	N/A		2	1
	Son or daughter	N/A	N/A		41	15
	Other relation	N/A	N/A		1	0
	Friend	N/A	N/A		1	0
	Other	N/A	N/A		5	2
	Total	N/A	N/A		270	100
	Prefer not to say	N/A	N/A		1	
	Missing	N/A	N/A		175	
Parent’s/carer’s living arrangements	Lives with patient	N/A	N/A		244	91
	Does not live with patient	N/A	N/A		24	9
	Total	N/A	N/A		268	100
	Prefer not to say	N/A	N/A		3	
	Missing	N/A	N/A		175	
Geographical region	South East of England	65	13		35	13
	South West of England	61	12		26	10
	Scotland	60	12		21	8
	London	52	10		26	10
	North West of England	51	10		34	13
	East of England	42	8		17	6
	Wales	39	8		9	3
	Yorkshire	35	7		16	6
	West Midlands	31	6		48	18
	East Midlands	24	5		17	6
	North East and Cumbria	23	5		14	5
	Northern Ireland	15	3		1	0
	Other	8	2		7	3
	Total	506	100		271	100
	Prefer not to say	4			0	
	Missing	250			175	
Ethnic group	White	473	94		245	92
	Non-white	20	4		15	6
	Other	8	2		5	2
	Total	501	100		265	100
	Prefer not to say	9			6	
	Missing	250			175	
Educational attainment	No formal qualifications	18	4		6	2
	O level or GCSE	68	14		41	16
	ONC or BTEC	21	4		14	5
	A level ('Higher' in Scotland)	35	7		26	10
	Higher education qualification	102	21		40	16
	Degree or higher degree	252	51		130	51
	Total	496	100		257	100
	Prefer not to say	14			14	
	Missing	250			175	

#### Healthcare professionals

Healthcare professionals were recruited from all regions in the UK. Participants reported mixed clinical expertise in rare diseases, with 56% (n = 136) reporting they have clinical expertise in rare diseases and 44% (n = 107) reporting that they do not have clinical expertise in rare diseases; perhaps demonstrating that many healthcare professionals working with rare diseases may not be rare disease specialists but rather seeing rare disease patients within their usual healthcare professional role.

Healthcare professionals reported being involved in a range of different areas. The most commonly reported healthcare professional activity was providing information/signposting or counselling (n = 189; 75%). A range of healthcare professional roles were reported, including hospital doctors (n = 78, 31%), nurse/midwives (n = 39, 16%) and allied healthcare professionals (n = 28, 11%). See Table 3 for more information.

**Table 3:** Sample characteristics of healthcare professionals

Characteristic	Response	Number (n = 251)	% of respondents
Geographical region	South East of England	9	7
	South West of England	12	5
	Scotland	6	2
	London	34	14
	North West of England	66	26
	East of England	6	2
	Wales	1	1
	Yorkshire	4	2
	West Midlands	25	10
	East Midlands	11	4
	North East & Cumbria	7	3
	Northern Ireland	1	1
	Other	4	2
	Total	186	100
	Prefer not to say	0	
	Missing	65	
Clinical expertise in rare diseases	Yes	136	56
	No	107	44
	Total	243	100
	Missing	8	
Areas of work with patients with rare conditions^a^	Diagnosing condition	148	59
	Providing information/signposting, or counselling	189	75
	Long-term care following diagnosis	166	66
	Long-term care in the absence of a diagnosis	139	55
Healthcare professional role	Hospital doctor	78	34
	Nurse/midwife	39	17
	Allied health professional	28	12
	Clinical academic	24	10
	GP/community doctor	12	5
	Manager	7	3
	Public health professional	5	2
	Health informaticist	4	2
	Psychological therapist	3	1
	Patient representative	3	1
	Pharmacist	1	0
	Commissioner	1	0
	Other	26	11
	Total	231	100
	Missing	20	

^a^Respondents could work in more than one area so the numbers sum to more than 251

### Findings

#### What levels of access to combinations of elements and individual elements of care coordination (care coordinators, specialist centres, care plans) do patients and carers report?

##### Access to combinations of care coordination elements

Findings highlighted that the most common ‘combination’ of elements of care coordination reported by patients (n = 326, 54%) and parents/carers (n = 115, 33%) was having access to none of the elements of care coordination (care coordinator, care plan and specialist centre). A small minority of participants reported having access to all three of the elements (care coordinator, care plan, specialist centre) (n = 14, 2% patients; n = 17, 5% parents/carers), or a combination of two elements, such as care coordinator and specialist centre (n = 30, 5% patients; n = 5, 1% parents/carers). See Table 4 for more details.

**Table 4:** Patients’ and carers’ reported access to combinations of care coordination elements

Combination	Reported access to combinations of care coordination elements (care coordinator^a^, care plan^b^, specialist centre^c^)	Patients		Parents/carers	
		Number	%	Number	%
1	None of the elements *(no care coordinator, no care plan, no specialist centre)*	326	54	115	33
2	Specialist centre only	169	28	66	19
5	Care coordinator + specialist centre	30	5	5	1
6	Care coordinator only	24	4	9	3
4	Care plan + specialist centre	22	4	42	12
3	Care plan only	17	3	80	23
7	All of the elements *(care coordinator, care plan, specialist centre)*	14	2	17	5
8	Care coordinator + care plan	2	0	14	4
	Total	604^d^	100	348^d^	100

Combinations are ranked in order of prevalence for patients

^a^For patients the question is ‘Do you have a formal care coordinator? Yes/No/Unsure’; for parents/carers it is’Does the person you care for have a formal care coordinator? Yes/No/Unsure’

^b^For patients the question is ‘Do you have a care plan relating to your rare condition? Yes/No/Unsure’; for parents/carers it is ‘Does the person you care for have a care plan relating to their rare condition? Yes/No/Unsure’

^c^For patients the question is ‘Is there a specialist centre available for you? Yes/No/Unsure”; for parents/carers it is ‘Is a specialist centre available for the person you care for? Yes/No/Unsure’

^d^Respondents who responded ‘Unsure’ to any of these questions or who did not respond to all of these questions were excluded (156 patients, 98 parents/carers)

##### Access to individual elements of care coordination

Findings on access to individual elements of care coordination (care coordinators, specialist centres and care plans), as reported by patients, parents/carers are summarised in Table 5.

**Table 5:** Patients’ and carers’ reported levels of access to three elements of care coordination (care coordinators, specialist centres, care plans)

Element of care coordination	Question	Response	Patients (n = 760)		Parents/carers (n = 446)	
			Number	%	Number	%
Care coordinators	Do you (patients)/the person you care for (parents/carers) have a formal care coordinator?	Yes	92	12	62	14
		No	570	77	325	76
		Unsure	76	10	43	10
		Total	738	100	430	100
		Missing	22		16	
	Is the formal care coordinator employed specifically for the role (or do they coordinate care as part of another role, e.g., GP, specialist nurse?) *(if patients/parents or carers have a formal care coordinator)*	Yes	33	36	19	31
		No	51	56	38	61
		Unsure	7	8	5	8
		Total	91	100	62	100
		Not applicable	646		368	
		Missing	23		16	
	What is the formal care coordinator’s role? *(if formal care coordinators role is part of another role)*	Hospital doctor	25	49	9	24
		GP	14	27	5	13
		Specialist nurse	7	14	9	24
		Other	3	6	5	13
		Practice or community nurse	2	4	3	8
		Community paediatrician	0	0	3	8
		Palliative Care specialist	0	0	2	5
		Charity or patient support group representative	0	0	1	3
		Physiotherapist	0	0	1	3
		Genetic counsellor	0	0	0	0
		Total	51	100	38	100
		Not applicable	686		392	
		Missing	23		16	
	Which items are managed by the formal care coordinator? *(if applicable)*	Liaising between healthcare professionals	69	75	45	73
		Scheduling appointments	56	64	23	37
		Contact for emergency or acute episodes	35	38	21	34
		Updating care plan	32	35	23	37
		Ensuring availability of health records at appointments	25	27	8	13
		Liaising with patient to coordinate multi-disciplinary clinics	21	23	18	29
		Advocating on patient’s behalf	16	17	19	31
		Out of hours contact	16	17	3	5
		Coordinating transitions of care	13	14	17	27
		Liaising between health and non-healthcare professionals (e.g. social worker, homecare)	11	12	26	42
		Arranging respite care	1	1	7	11
		Total	92		62	
		Not applicable	668		384	
Specialist centres	Is there a specialist centre available for you (patients)/the person you care for? (parents/carers)?	Yes	235	39	130	37
		No	250	41	168	48
		Unsure	119	20	50	14
		Total	604	100	348	100
		Missing	156		98	
	Do you (patients) or they (parents/carers) attend a specialist centre? *(if specialist centre available)*	Yes	196	83	114	88
		No	35	15	14	11
		Unsure	4	2	2	2
		Total	235	100	130	100
		Not applicable	369		218	
		Missing	156		98	
	Which healthcare professionals are seen at the specialist centre? *(if participants attend specialist centre)*	Doctors who are expert in rare or undiagnosed conditions	166	85	86	75
		Specialist nurse	123	63	74	65
		Doctors who are expert in aspects of health affected (e.g. neurologist)	111	57	72	63
		Physiotherapist	32	16	35	31
		Psychologist	30	15	29	25
		Dietician	22	11	36	32
		Genetic counsellor	9	5	17	15
		Occupational therapist	8	4	17	15
		Care coordinator	7	4	10	9
		Behavioural therapist	1	1	3	3
		Community paediatrician			8	7
		Speech and language therapist			19	17
		Other	30	15	13	11
		Total	196		114	
	Which services are provided by the specialist centre?	Appointments with an expert in rare conditions	170	87	83	73
		Appointments to see different types of healthcare professionals at the centre	118	60	80	70
		Multiple appointments during a single visit	90	46	62	54
		Diagnostic and screening procedures	86	44	53	46
		Access to patient support groups or charities	79	40	35	31
		Access to research opportunities	69	35	41	36
		Contact for acute or emergency episodes	52	27	46	40
		Non-urgent, out-of-hours contact	50	26	35	31
		Appointments which are not in-person (e.g. virtual or telephone appointments)	44	22	23	20
		Support during emergency admissions	32	16	31	27
		Support with routine admissions	31	16	31	27
		Appointments to see non-healthcare professionals (e.g. social worker)	26	13	19	17
		Extended hours for appointments	12	6	7	6
		Other	12	6	4	4
		Total	196		114	
Care plans	Do you (patients)/the person you care for (parents/carers) have a care plan relating to their rare condition?	Yes	59	10	159	44
		No	478	78	165	46
		Unsure	76	12	37	10
		Total	613		361	
		Not stated	147		85	
	Who is primarily responsible for keeping the care plan up-to-date?	The patient	15	27	1	1
		Hospital doctor	15	27	5	3
		Shared responsibility between professionals	8	14	19	12
		No one holds responsibility	5	9	9	6
		Specialist nurse	4	7	8	5
		Formal care coordinator	2	4	2	1
		GP	2	4	0	0
		Genetic counsellor	1	2	0	0
		The carer	0	0	59	37
		Practice or community nurse	0	0	5	3
		Community paediatrician	0	0	2	1
		Other	4	7	49	31
		Total	56	100	159	100
		Not applicable	554		202	
		Missing	150		85	
	Were you (patients) or the person you care for (parents/carers) involved in developing the care plan for your needs?	Yes	36	64	135	85
		No	14	25	19	12
		Unsure	6	11	5	3
		Total	56	100	159	100
		Not applicable	554		202	
		Missing	150		85	
	What is addressed in the care plan?	General information and a medical summary	51	91	142	89
		An assessment of current health needs	39	70	117	74
		Scheduled reviews of the care plan	20	36	65	41
		Plan of care for emergency or acute episodes	19	34	77	48
		Out of office (non-urgent) contacts	14	25	33	21
		An assessment of current non-health needs (e.g. social care)	11	20	80	50
		Documented health goals	11	20	45	28
		Transition planning for changes in care	8	14	19	12
		Other	2	4	21	13
		Total	56		159	
	What are the 3 most useful items that should be included in a care plan?	An assessment of current health needs	485	64	273	61
		General information and a medical summary	459	60	259	58
		Plan of care for emergency or acute episodes	459	47	196	44
		Scheduled reviews of the care plan	173	23	79	18
		Out of office hours (non-urgent) contacts	108	14	51	11
		An assessment of current non-health needs (e.g. social care)	97	13	108	24
		Documented health goals	94	12	51	11
		Transition planning for changes in care	46	6	45	10

**Care coordinators** Findings indicated that access to a formal care coordinator was infrequently reported by patients (n = 92, 12%) and parents/carers (n = 62, 14%); with the majority of patients (n = 570, 77%) and carers (n = 325, 76%) reporting that they do not have a formal care coordinator.

Of those who reported having a formal care coordinator (n = 92 patients, 62 parents/carers), 36% (n = 33) of patients and 31% (n = 19) parents/carers reported that the formal care coordinator was employed specifically for the role. However, the majority of participants (patients: n = 51, 56%; carers: n = 38, 61%) reported that a healthcare professional coordinated their care as part of another role. Examples of the most frequent roles reported were hospital doctors (patients: n = 25, 49%, carers: n = 9, 24%), GPs (patients: n = 14, 27%, carers: n = 5, 13%), specialist nurses (patients: n = 7, 14%; carers: n = 9, 24%).

Participants reported that the most common roles managed by the formal care coordinator included: liaising between healthcare professionals (patients: n = 69, 75%, carers: n = 45, 93%), scheduling appointments (patients: n = 56, 64%, carers: n = 23, 37%), being a contact for emergency or acute episodes (patients: n = 35, 38%, carers: n = 21, 34%), updating care plans (patients: n = 32, 35%, carers: n = 23, 37%), ensuring availability of health records (patients: n = 25, 27%, carers: n = 8, 13%) and liaising with patients to coordinate multidisciplinary clinics (patients: n = 21, 23%, carers: n = 18, 29%). See Table 5 for more information.

**What roles do participants prefer a formal care coordinator to manage, versus the patient/carer?** A majority of respondents from all three groups (patients, carers, healthcare professionals) reported preferences for all care activities shown in Table 6 to be managed by a formal care coordinator, except for scheduling appointments—which all groups preferred to be managed by the patient and carer.

**Table 6:** Preferences for activities managed by the patient/carer or formal care coordinator

Question	Response	Patients (n = 654)^a^				Parents/carers (n = 391)^b^				Healthcare professionals (n = 208)^c^			
		Preference to be managed by Patient/carer		Preference to be managed by Formal care coordinator		Preference to be managed by Patient/ carer		Preference to be managed by Formal care coordinator		Preference to be managed by Patient/ carer		Preference to be managed by Formal care coordinator	
		Number	%	Number	%	Number	%	Number	%	Number	%	Number	%
Which items would you prefer to be managed by the patient/carer or formal care coordinator?	Scheduling appointments	413	63	241	37	284	75	107	25	120	58	88	42
	Liaising between healthcare professionals	133	20	521	80	76	19	315	81	9	4	199	96
	Liaising between health and non-healthcare professionals (e.g. social worker, homecare)	178	27	476	73	129	33	262	67	21	10	187	90
	Updating care plan	166	25	488	75	137	35	254	65	27	13	181	87
	Ensuring availability of health records at appointments	121	19	533	81	59	15	332	85	24	12	184	88
	Coordinating transitions of care	107	16	547	84	66	17	325	83	7	3	201	97
	Liaising with patient to coordinate multi-disciplinary clinics	121	19	533	81	93	24	298	76	20	10	188	90
	Arranging respite care	158	24	496	76	146	37	245	63	61	29	147	71

^a^106 missing, ^b^55 missing, ^c^43 missing

**Specialist centres** Not having a specialist centre available (patients: n = 250, 41%; parents/carers: n = 168, 48%) was more commonly reported by patients and carers than having a specialist centre available (patients: n = 235, 39%, carers: n = 130, 37%).

Of those who had a specialist centre available, approximately a third of patients (n = 196/604, 32%) and carers (n = 114/348, 33%) reported attending the specialist centre.

Participants who attend specialist centres (n = 196) reported seeing a range of different healthcare professionals, including: doctors who are experts in rare/undiagnosed conditions (patients: n = 166, 85%, carers: n = 86, 75%), specialist nurses (patients: n = 124, 63%, carers: n = 74, 65%), doctors who are experts in specific aspects of their health (patients: n = 111, 57%, carers: n = 72, 63%), and physiotherapists (patients: n = 32, 16%; carers: n = 35, 31%). Participants reported a range of services being delivered at specialist centres, including appointments with rare condition experts (patients: n = 170, 87%, carers: n = 83, 73%), appointments with different healthcare professionals (patients; n = 118, 60%, carers: n = 80, 70%), having multiple appointments during one visit (patients: n = 90, 46%, carers: n = 62, 54%), diagnostic and screening procedures (patients: n = 86, 44%, carers: n = 53, 46%), and access to patient support groups or charities (patients: n = 79, 40%, carers: n = 35, 31%). See Table 5 for more information.

**Care plans** Care plans were more frequently reported by parents and carers (n = 159, 44%) than by patients (n = 59, 10%). However, a majority of patients (n = 478, 78%) and almost half of parents/carers (n = 165, 46%) reported not having a care plan. See Table 5 for more details.

Of those who had a care plan, responses differed between patients and carers on who was primarily responsible for keeping care plans up to date. Patients most frequently reported that responsibility was with: the patient (n = 15, 27%), the hospital doctor (n = 15, 27%) or a shared responsibility between professionals (n = 8, 14%). Carers most frequently reported that the responsibility was with the carer (n = 59, 37%), or a shared responsibility between professionals (n = 19, 12%). Many of the patients and carers reported being involved in developing their care plans (patients: n = 36, 64%, carers: n = 135, 85%).

Patients and carers reported a range of aspects that are addressed in their care plans, including general information and medical summaries (patients: n = 51, 91%, carers: n = 142, 89%), an assessment of health needs (patients: n = 39, 70%, carers: n = 117, 74%), a plan of care for emergency or acute episodes (patients: n = 19, 34%, carers: n = 77, 48%) and an assessment of current non-health needs (patients: n = 11, 20%; carers: n = 80, 50%). Patients and carers felt that the three most useful items to be included in a care plan were: an assessment of current health needs (patients: n = 485, 64%, carers: n = 273, 61%), general information and a medical summary (patients: n = 459, 60%, carers: n = 259, 58%) and a plan of care for emergency or acute episodes (patients: n = 459, 47%, carers: n = 196, 44%).

#### What levels of access to three elements of care coordination (care coordinators, specialist centres, care plans) do healthcare professionals report?

Findings on access to individual elements of care coordination (care coordinators, specialist centres and care plans), as reported by healthcare professionals are summarised in Table 7.

**Table 7:** Healthcare professionals’ reported level of access to three elements of care coordination (care coordinators, specialist centres, care plans)

Element of care coordination	Question	Response	Number (n = 251)	%
Care coordinator	Do the majority of your patients have a formal care coordinator?	Yes	82	35
		No	118	51
		Unsure	33	14
		Total	233	100
		Missing	18	
	Is the formal care coordinator employed specifically for the role (or do they coordinate care as part of another role, e.g., GP, specialist nurse?) *(If formal care coordinator available)*	Yes	15	19
		No	61	75
		Unsure	5	6
		Total	81	100
		Not applicable	151	
		Missing	19	
	What is the formal care coordinator’s main role? *(if care coordinator role is part of another role)*	Hospital doctor	16	26
		GP	6	10
		Specialist nurse	19	31
		Other	6	10
		Practice or community nurse	0	0
		Community paediatrician	13	21
		Palliative Care specialist	0	0
		Charity or patient support group representative	0	0
		Physiotherapist	0	0
		Genetic counsellor	1	2
		Total	61	100
		Not applicable	171	
		Missing	19	
	Which items are managed by the formal care coordinator?	Liaising between healthcare professionals	75	75
		Scheduling appointments	41	41
		Contact for emergency or acute episodes	42	42
		Updating care plan	55	55
		Ensuring availability of health records at appointments	34	34
		Liaising with patient to coordinate multi-disciplinary clinics	48	48
		Advocating on patient’s behalf	63	63
		Out of hours contact	21	21
		Coordinating transitions of care	60	60
		Liaising between health and non-healthcare professionals (e.g. social worker, homecare)	69	69
		Arranging respite care	36	36
		Total	100	
		Not applicable	151	
	What are the main factors that determine whether someone with a rare condition will have access to a formal care coordinator?	Complexity of disease	124	49
		Availability of care coordinators	124	49
		Extent of patient’s need for support	113	45
		Budgetary constraints	87	35
		Request of patient / carer / family	80	32
		Caseload of healthcare professionals involved	76	30
		Patient’s existing support system (number and role of carers)	67	27
		Distance from specialist centre	57	23
		Unsure	29	12
		Total	251	100
Specialist centres	Is there a specialist centre available for the majority of your patients with rare conditions?	Yes	122	60
		No	61	30
		Unsure	22	11
		Total	205	100
		Missing	46	
	Which healthcare professionals are seen at the specialist centre? *(if specialist centre available)*	Doctors who are expert in rare or undiagnosed conditions	94	64
		Specialist nurse	98	67
		Doctors who are expert in aspects of health affected (e.g. neurologist)	94	64
		Physiotherapist	65	44
		Psychologist	67	46
		Dietician	66	45
		Genetic counsellor	76	52
		Occupational therapist	55	37
		Care coordinator	29	20
		Behavioural therapist	13	9
		Community paediatrician	22	15
		Speech and language therapist	55	37
		Other	30	20
		Total	147	
	Which services are provided by the specialist centre?	Appointments with an expert in rare conditions	92	63
		Appointments to see different types of healthcare professionals at the centre	92	63
		Multiple appointments during a single visit	75	51
		Diagnostic and screening procedures	89	61
		Access to patient support groups or charities	86	59
		Access to research opportunities	92	63
		Contact for acute or emergency episodes	63	43
		Non-urgent, out-of-hours contact	35	24
		Appointments which are not in-person (e.g. virtual or telephone appointments)	61	42
		Support during emergency admissions	62	42
		Support with routine admissions	53	36
		Appointments to see non-healthcare professionals (e.g. social worker)	34	23
		Extended hours for appointments	14	10
		Other	5	3
		Total	147	
	What are the main reasons why patients with rare conditions might choose not to use specialist centres?	Distance to travel to specialist centre	179	71
		Cost of travel to specialist centre	166	66
		Physical difficulty in travelling to specialist centre	159	63
		Patient is satisfied with quality of care provided locally	87	35
		Length of time between appointments at specialist centre	81	32
		Perceived lack of benefit from the specialist centre	60	24
		Length of appointment times at specialist centre	41	16
		Other	39	16
		Total	251	
Care plans	Do you use care plans as a means to document care for patients with rare conditions?	Yes	82	40
		No	105	51
		Unsure	20	10
		Total	207	
		Not stated	44	
	Who is primarily responsible for keeping the care plan up-to-date?	The patient	4	5
		Hospital doctor	7	9
		Shared responsibility between professionals	20	25
		No one holds responsibility	5	6
		Specialist nurse	17	21
		Formal care coordinator	6	7
		GP	2	2
		Genetic counsellor	0	0
		The carer	0	0
		Practice or community nurse	1	1
		Community paediatrician	5	6
		Other	14	17
		Total	81	100
		Not applicable	125	
		Missing	45	
	What are the 3 most useful items that should be included in a care plan?	An assessment of current health needs	149	59
		General information and a medical summary	155	62
		Plan of care for emergency or acute episodes	161	64
		Scheduled reviews of the care plan	19	8
		Out of office hours (non-urgent) contacts	32	13
		An assessment of current non-health needs (e.g. social care)	51	20
		Documented health goals	24	10
		Transition planning for changes in care	24	10

##### Care coordinators

Around half 51% (n = 118) of healthcare professionals reported that the majority of their patients do not have access to a formal care coordinator, in comparison to 35% (n = 82) who do. Some healthcare professionals (14%, n = 33) were unsure whether their patients had a formal care coordinator.

Of those healthcare professionals who reported that their patients have a formal care coordinator (n = 82), 19% (n = 15) reported that the formal care coordinator was employed specifically for the role. However, the majority of healthcare professionals (n = 61, 75%) reported that a healthcare professional coordinates their care as part of another role. Examples of the most frequent roles reported were hospital doctors (n = 16, 26%), specialist nurses (n = 19, 31%) and community paediatricians (n = 13, 21%).

Healthcare professionals reported that the most common roles managed by the formal care coordinator included: liaising between healthcare professionals (n = 75, 75%), liaising between health and non-healthcare professionals (n = 69, 69%), advocating on the patient’s behalf (n = 63, 63%), coordinating transitions of care (n = 60, 60%), and updating care plans (n = 55, 55%). Healthcare professionals reported that the main factors determining whether someone will have access to a formal care coordinator included: the complexity of their disease (n = 124, 49%), the availability of care coordinators (n = 124, 49%), the extent of the patient’s need for support (n = 113, 45%), and budgetary constraints (n = 87, 32%). See Table 6 for more information.

##### Specialist centres

In comparison to the patient/carer findings, the majority of healthcare professionals reported that there is a specialist centre available for the majority of their patients with rare conditions (n = 122, 60%). However, specialist centres were not available for all patients, with 30% (n = 61) of healthcare professionals reporting that their patients did not have access to one.

Healthcare professionals reported that a range of professionals are seen at the specialist centre, including: doctors who are experts in rare/undiagnosed conditions (n = 94, 64%), specialist nurses (n = 98, 67%) doctors who are experts in specific aspects of their health (n = 94, 64%), psychologists (n = 67, 46%), dietitian (n = 66, 45%) and physiotherapists (n = 65, 44%). Healthcare professionals reported a range of services being delivered at specialist centres, including appointments with rare condition experts (n = 92, 63%), access to research opportunities (n = 92, 63%), and diagnostic and screening procedures (n = 89, 61%).

The most frequently reported reasons given by healthcare professionals for patients not using specialist centres included the distance (n = 179, 71%), cost (n = 166, 66%) and physical difficulties (n = 159, 63%) associated with travelling to specialist centres. See Table 6 for more information.

##### Care plans

More healthcare professionals reported that they do not use care plans to document care for patients with rare conditions (n = 105, 51%), than those who do use care plans (n = 82, 40%).

Of those who reported using a care plan (n = 81), healthcare professionals most frequently reported that responsibility was: a shared responsibility between professionals (n = 20, 25%), the responsibility of a specialist nurse (n = 17, 21%), or other (n = 14, 17%).

Healthcare professionals felt that the three most useful items were: a plan of care for emergency or acute episodes (n = 161, 64%), general information and a medical summary (n = 155, 62%), and an assessment of current health needs (n = 149, 59%). See Table 6 for more information.

## Discussion

### Key findings

Our national UK survey of patients, parents/carers and healthcare professionals highlighted that most patients and families report not having a formal care coordinator, not attending a specialist centre or not having access to a care plan. Additionally, over half of patients and a third of carers/parents did not have access to any of these elements of care coordination. More parents and carers reported that they had a care plan than adult patients; suggesting that perhaps care plans are more frequent for families of children with rare conditions than adult patients. Additionally, healthcare professionals reported access to elements of care coordination more frequently (e.g. almost two thirds reported that there is a specialist centre available).

### How the findings relate to previous research

Our findings support existing evidence which suggests that care coordination for rare conditions is currently limited in practice [5]. Additionally, the findings from this study extend findings from the wider CONCORD study. For example, the CONCORD scoping review found that care coordination includes a range of components; of which care plans, specialist centres and care coordinators were integral [4]. Similarly, these elements of care coordination were found to be key aspects of the CONCORD taxonomy [24, 25]. This study therefore illustrates a need to improve patients access to care coordinators, specialist centres and care plans to improve care coordination. However, care coordination is not ‘one size fits all’, with models of care coordination needing to consider a range of factors including where the patient lives, and whether they are able to support coordination of their own care [25]. Previous research highlights that more care coordination is needed in complex situations (e.g. increased clinical complexity) [26]. However, it is not clear whether those patients with increased clinical complexity and need are currently experiencing better coordination. These findings support and emphasise the need to focus on improving care coordination for rare conditions in future; as illustrated in current policy documents [8–13].

Previous research highlights the importance of care coordinators for rare conditions [4–6]. However, findings from our survey indicated that care coordinators were infrequently reported by patients and carers. Our study demonstrated that healthcare professionals were more aware of care coordinators being in place for patients with rare conditions. These findings support previous research which suggests that only a small number of patients with rare conditions may receive support from a care coordinator and that patients and carers are currently holding the responsibility of coordinating care. Our findings therefore contrast with findings that care coordinators have been widely used for adults and children with chronic conditions and mental health conditions in the UK and worldwide [27–30].

Existing evidence highlighted the importance of specialist centres for people living with rare conditions [24, 25, 31]. Our findings indicated that whilst many healthcare providers are aware of specialist centres for rare conditions for the majority of their patients, not all patients and carers are aware of, or have access to, a specialist centre for their rare condition. One possible explanation for this is that not all rare conditions can have specialist centres. There may also be bias in our sample, in that the healthcare professionals who were interested in taking part may be more engaged in working with rare conditions and therefore see patients who do have access to specialist centres. Additionally, another explanation is that patients and carers may not be getting referred to specialist centres as needed.

Previous research has also highlighted a need for care plans to support coordination of care for rare conditions [4, 24]. Our findings extend previous research by highlighting potential gaps in the use of care plans with more parents/carers reporting having care plans than adult patients. This is likely to be due to the established system of education, health and care plans (EHCPs) for children in the UK with special educational needs and/or disabilities.

### Strengths and limitations

This study achieved a wide sample of different types of respondents (patients/carers/healthcare professionals), rare conditions and geographical areas across the UK. One strength was that we did not limit the type of rare condition. This enabled us to identify as many experiences of care coordination as possible.

There is limited evidence on the total number of people living with a rare condition in the UK, and their characteristics such as gender, age distribution, ethnicity, level of education and socio-economic status are unknown. Therefore, it was not possible to explore how representative our sample was of the wider rare disease community. For example, it is possible that our sample may have been over representative of certain conditions (e.g. sarcoidosis). We were unable to track response rates as surveys were distributed via overlapping distribution routes.

A further limitation is that we used convenience sampling (through community groups and NHS organisations) to identify participants. Therefore, some groups were underrepresented in our sample (e.g. those who don’t have links to the patient organisations or use the selected NHS organisations). Additionally, those who may not have a computer or email address, or who have lower digital health literacy may be under-represented. No formal steps were taken to eliminate potential dual-parent responses, therefore it is possible that both parents may have completed the survey and reported similar experiences.

Our sample also had a high proportion of female respondents (patients and parents/carers). This is consistent with other surveys within the rare disease community [32]. However, this may partially be explainable for the parent / carer part of the sample by women potentially being more likely to be the main carer for their child, thus more likely to identify as someone that should complete the survey.

### Implications

Our findings have a range of implications for policy and practice. In particular, they illustrate substantial gaps in care coordination across the UK. Better care coordination could include: (1) improving access to care coordinators, (2) improving access to specialist centres (by establishing new centres for under-served conditions, and facilitating referral to existing centres), and (3) working to put agreed care plans in place for adults and children living with rare conditions.

### Further research

This study highlights how patients, families and healthcare professionals experience care coordination currently and demonstrate gaps in provision of care coordination elements. Whilst research has considered preferences of care coordination [33] and models of care coordination for rare conditions [24, 25], further research is needed to evaluate the effectiveness and cost of models of care coordination in practice. Additionally, further research should explore why different respondents have different perceptions of care coordination.

## Conclusions

The findings of this study highlight that care for people affected by rare diseases is generally not well coordinated in the UK, with limited access to care coordinators, specialist centres and care plans. Different respondents had different perspectives of care coordination, with healthcare professionals reporting that their own patients had better access to care coordination than patients and parents/carers in the sample; and more parents/carers reporting access to care plans than affected adults. Better understanding of these issues can inform how care coordination might be improved and centred around the needs and preferences of patients and families affected by rare conditions.

### Supplementary Information

**Additional file 1.** Copy of patient survey.

**Additional file 2.** Copy of parent/carer survey.

**Additional file 3.** Copy of healthcare professional survey.

## Data Availability

All data requests should be submitted to the corresponding author for consideration. Access to available anonymised data may be granted following review.
