# Supplementary material for: Experiences of coordinated care for people in the UK affected by rare diseases: cross-sectional survey of patients, carers, and healthcare professionals
Source: Orphanet J Rare Dis. 2023 Nov 23;18:364. doi: 10.1186/s13023-023-02934-9 (PMC10668407; doi:10.1186/s13023-023-02934-9)
Supplement: Supplementary file 1 — Additional file 1. Copy of patient survey. [file 13023_2023_2934_MOESM1_ESM.docx]

**Participant Information Sheet for CONCORD Survey**

**Title of Study:** COORDINATED CARE OF RARE DISEASES (CONCORD)

**Information about our survey:**

Thank you for taking the time to look at our survey.

We are interested in learning about the health care experiences of people with rare conditions, specifically about how care is coordinated when people have a range of different needs.

This survey is part of a larger study, called Coordinated Care of Rare Diseases (CONCORD), which is sponsored by University College London (UCL), funded by the UK National Institute for Health Research (NIHR) and approved by an independent ethics committee:

Research Ethics Committee ID Number: 19/LO/0250.

**Name and Contact Details of the Researcher(s): 
Emma Hudson:** [**e.hudson@ucl.ac.uk**](mailto:e.hudson@ucl.ac.uk)

**Name and contact details of company providing survey:**

**Accent, Alison Lawrence: Alison.lawrence@accent-mr.com**

**Name and Contact Details of the Principal Researcher: 
Stephen Morris:**[**sm2428@medschl.cam.ac.uk**](mailto:sm2428@medschl.cam.ac.uk)

**What is the study about?**

The COordiNated Care of Rare Diseases (CONCORD) study aims to explore how care for rare diseases is coordinated in the UK and how patients, families and healthcare professionals would like them to be coordinated. You are being invited to take part in a survey, which is part of the overall CONCORD study. The findings will help us understand more about care coordination and preferences for care coordination. This study is being conducted by researchers in the Department of Applied Health Research at University College London.

**Can I participate?**

You can participate if:

- you are a patient with a rare, ultra-rare, or undiagnosed condition **or**
- you have experience of caring for a patient with a rare, ultra-rare, or undiagnosed condition **or**
- you are a health professional who has experience in caring for patients that fit this definition.

We are aiming to collect around 1500 responses.

**Do I have to take part?**

No. Taking part in this survey is voluntary. If you choose not to take part, it will not affect the care you receive from the NHS in any way.

**What would taking part involve?**

If you decide to take part, you will be asked to complete a survey about care coordination. The first part of the survey asks you to tell us about your experiences of care coordination and your preferences for how care should be coordinated. The second part of the survey will ask you to make a choice between two different scenarios of care coordination. This is a really helpful way of showing us what your priorities are for these different aspects of care coordination. The survey will be managed by a professional survey company who will have a service level agreement with UCL outlining data protections.

You can complete this survey online, or if you’d prefer, you can request to complete the survey in hardcopy, electronically, or over the phone. Requests for hardcopies, electronic copies, or completion over the phone can be made directly with the professional survey company, Accent, by emailing CONCORD@accent-mr.com or calling their FREEPHONE number 0800 084 2783). Your consent to participate in this survey will be implied if you submit an online, electronic, or hardcopy version of the survey. Your consent will be confirmed verbally if you choose to take the survey over the phone.

**How long will it take to complete the survey?**

The survey takes most people up to 30 minutes to complete but if you are doing it online you can take a break at any point and finish it when you are ready by using the same link to the website.

**What are the possible disadvantages and risks of taking part?**

The survey will take some time to complete but there are no other foreseeable disadvantages or risks of taking part in this survey.

**What are the possible benefits of taking part?**

There are no immediate benefits for those people participating in the project, however, it is hoped that this study will support improvements in how care is coordinated for people with rare conditions across the NHS in England.

**What if something goes wrong?**

If you have any queries about the questionnaire, please contact Emma Hudson e.hudson@ucl.ac.uk. If you experience any technical difficulties with filling in the questionnaire, please call the survey company on FREEPHONE helpline number *0800 084 2783* or email CONCORD@accent-mr.com. If you have any complaints about this study, please contact the Principal Researcher, Prof Stephen Morris at [sm2428@medschl.cam.ac.uk](mailto:sm2428@medschl.cam.ac.uk). Should you feel your complaint has not been handled to your satisfaction, you can contact the UCL Research and Development office at [uclh.randd@nhs.net](mailto:uclh.randd@nhs.net).

UCL holds insurance against claims from participants for harm caused by their participation in this research. Participants may be able to claim compensation if they can prove that UCL has been negligent.

**Will my taking part in this project be kept confidential?**

We will not tell anyone that you have taken part in this survey. If you need an electronic copy or hardcopy of this survey, or decide to complete it over the phone with the survey company, the survey company will need to have your contact details, but these details will only be used for this purpose and will not be shared with anyone else. All information collected during the study will be kept strictly confidential. The results of the survey will be used in written reports, academic publications, conference publications or any other material produced for the study. We will not use your name and will make every effort to protect your identity.

**Limits to confidentiality**

Confidentiality will be respected subject to legal constraints and professional guidelines.

**How will my personal information be handled?**

The information you provide as part of the survey will be stored and handled by a professional survey distribution company with whom UCL has a service level agreement outlining data protections. The survey company will process your answers in confidence. They will then send your answers to researchers at UCL to be analysed. The survey company and UCL will store your responses in line with the Data Protection Act 1998 and new General Data Protection Regulation (GDPR). Any of your identifiable data we collect will be stored securely for up to three years after the end of the project and then destroyed securely. Anonymised data will be archived for 20 years. Your responses to the questionnaire will be processed only for the purposes required for this research project. No personal data will be transferred outside the European Economic Area (EEA). Data used in any publication will be fully anonymised.

If you are concerned about how your response data is being processed, please contact UCL in the first instance at data-protection@ucl.ac.uk. If you remain unsatisfied, you may wish to contact the Information Commissioner’s Office (ICO). Contact details, and details of data subject rights, are available on the ICO website at: https://ico.org.uk/for-organisations/data-protection-reform/overview-of-the-gdpr/individuals-rights/

**Data Protection Privacy Notice**By completing this questionnaire, you are giving your consent for your personal details and relevant health information to be held and analysed by a survey company and UCL for the purposes of this study only**.**

Please note:

- Your personal information will be handled securely and anonymised before analysis and before any publication.
- Your personal information will not be released by anyone working on the study unless required by law or where there is a clear overriding public interest.

University College London (UCL) is the sponsor for this study based in the United Kingdom. We will be using information from you in order to undertake this study and will act as the data controller for this study. This means that we are responsible for looking after your information and using it properly. UCL will keep identifiable information about you for three years after the study has finished.

Your rights to access, change or move your information are limited, as we need to manage your information in specific ways in order for the research to be reliable and accurate. To safeguard your rights, we will use the minimum personally-identifiable information possible. You can find out more about how we use your information by contacting the UCL Data Protection Office at [data-protection@ucl.ac.uk](mailto:data-protection@ucl.ac.uk) or by looking at the following website: <https://www.ucl.ac.uk/legal-services/privacy/participants-health-and-care-research-privacy-notice>

Your answers to the questionnaire will be processed for the purposes outlined in this notice. By clicking on the consent to participate button below you are agreeing that you have read and understood the information contained in this participant information sheet.

**START THE SURVEY**

# Completing the survey

For each question please choose the answer that best represents your views by ticking the corresponding box or selecting options from the lists provided. For some questions you may tick more than one box. Sometimes you will find the box you have ticked moves you on to other questions, this is so you do not spend time on questions that do not apply to you. If you make a mistake, simply change your selection to the correct answer. You can return to a previous question using the back arrow if you want to do so.

Some of the questions will ask you to recall details regarding your care, like the number of appointments you have, or how many healthcare professionals are involved in your care. If you cannot recall the exact information a close estimate is fine, so please try to complete the questions rather than leaving them unanswered.

There are comment boxes throughout the survey if you would like to give us more information to support your answer. If you do not wish to do so please leave these boxes blank.

The last section of the survey looks a bit different. You will see a number of questions where there are two options to choose from and we would like you to tell us which one you prefer. This is a really helpful way of showing us what your priorities are for these different aspects of care coordination.

Please do not put your name or address anywhere on the questionnaire.

Some of the terms we use in the survey might have different meanings to different people. We explain what we mean by these terms as they appear in the survey, but you can also find them all listed (here*)

Finally, thank you for participating in the survey, we really do appreciate the time you give up in order to share your experiences with us and we sincerely value all of the information provided.

THE CONCORD RESEARCH TEAM

Summary of survey terms **will appear when link is activated*

**Rare condition: In Europe a condition is considered to be a rare condition when it affects fewer than 5 in 10,000 people.**

**Ultra rare condition**: A disease is generally considered to be ultra-rare if it affects one patient per 50,000 people (or, fewer than 20 patients per million of population).

An **undiagnosed condition** is a condition which health professionals determine to be rare but tests have not provided a diagnosis. The condition might be complex and might have a genetic cause.

**NOTE: Unless specified in the question, the term rare condition denotes rare, ultra-rare and undiagnosed conditions.**

NOTE: for the purposes of the survey we use the term condition to refer to all rare conditions, diseases and syndromes.

A **specialist centre** is a centralised facility that enables patients to see a number of health professionals in one visit. Usually, the professionals at specialist centres will be experts in rare and undiagnosed conditions. Non-health professionals may also see patients at the same centre.

A **formal care coordinator** is a professional with a recognised role in helping patients and carers manage a range of needs between different professionals or across care settings. They may be a full-time coordinator or may coordinate care as part of their main job, such as a GP.

A **care plan** is a paper or electronic document which describes the health services and support that are needed and should be agreed between patients, carers and professionals. The care plan may be a single document or it may be part of another record which includes non-health services such as an Educational Care and Health Plan (ECHP).

**Care transitions** are permanent changes in care that can occur because of a change in needs such as transfer from child to adult services, or a change in circumstances such as relocation from one region to another.

# Suitability for survey

Please read the following statements carefully and tick the appropriate box.

If you answer NO for any of the statements, you are unfortunately not eligible to take part in the survey.

If you tick YES to all of the statements, you are eligible to take part in the survey.

|  | YES | NO |
| --- | --- | --- |
| I have a rare or undiagnosed condition. |  |  |
| I am 18 years old or over. |  |  |
| I am living in the UK and the majority of my health care is provided by the NHS. |  |  |
| I have read and understood the information regarding the survey. |  |  |
| I understand that by completing this questionnaire, I am giving consent for my personal details and relevant health information to be held and analysed by a survey company and UCL for the purposes of this study as outlined in the information provided. |  |  |
| I am aware of who I should contact if I wish to discuss any aspect of the study. |  |  |
| I give permission for the data I provide to be archived at University College London for up to three years after the end of the project. |  |  |

Did you find out about the survey from your NHS care provider?

*Tick only one:*

Yes

No *Go to Section A*

Unsure *Go to Section A*

If Yes, please tell us the name of the hospital or health care facility where you were told about the survey:

Care provider …………………………………………………………………………………………………………………………………………………………………………………………………………………………………………………………………………………………………………………………………………………………………………………………………………………………………………………………………………………………………………………

# Section A – Experience of diagnosis

In this section we would like you to tell us about your rare or undiagnosed condition.

*NOTE: If you have more than one rare condition, please answer in relation to* ***the rare condition which has the greatest impact on your health.***

Question 1

Have you been given a diagnosis of a rare condition by a health professional?

*Tick only one:*

Yes

No *Go to Question 4*

Unsure *Go to Question* 4

Question 2

What is your diagnosed rare condition?

Answer…………………………………………………………………………………………………………………………………………………………………………………………………………………………………………………………………………………………………………………………………………

**Question 3**

Has your diagnosis been confirmed by a genetic test?

*Tick only one:*

Yes *Go to Question 5*

No *Go to Question 5*

Unsure *Go to Question 5*

**Question 4**

Do you have a rare condition which is undiagnosed?

*Tick only one:*

Yes

No *Please check your eligibility to complete the survey*

Unsure *Please check your eligibility to complete the survey*

**Question 5**

1. How long after symptoms of the rare condition became apparent did you first seek medical advice?

*NOTE: (if immediately enter 0 years, 0 months)*

______months if known

_________years

Unsure Prefer not to say

1. Which medical professional was this initial contact with?

*Tick only one*

GP

Hospital doctor

Other, *provide details below*

1. IF YES AT Q4, GO TO QUESTION 6. How long after you became concerned about symptoms were you given a confirmed diagnosis of a rare condition?

……………………………………………………………………………………………………………………………………………………………………………………………………………………………………………………………………………………………………………………………………………………………………………………………………………………………………………………………………………………………………………………

_________years

______months, if known

Unsure Prefer not to say N/A undiagnosed condition *Go to Question 7*

**Question 6**

*NOTE: A* ***specialist centre*** *is a centralised facility that enables patients to see a number of health professionals in one visit. Usually, the professionals at specialist centres will be experts in rare and undiagnosed conditions. Non-health professionals may also see you at the same centre.*

1. Were you referred to a specialist centre before you received your diagnosis?

*Tick only one:*

Yes

No *Go to Question 7*

Unsure *Go to Question 7*

1. Were you diagnosed at a specialist centre?

*Tick only one:*

Yes

No *Go to Question 7*

Unsure *Go to Question 7*

1. If yes, how long after the first appointment at the specialist centre was diagnosis made?

_________years

_______months, if known

Unsure Prefer not to say

**Question 7**

How many times have you been given a diagnosis that turned out to be wrong (i.e., you were misdiagnosed)?

*Tick only one:*

*NOTE: if you were given a diagnosis related to symptoms (e.g., epilepsy or renal failure) which delayed further investigations to identify the underlying condition, this is also considered a misdiagnosis.*

*Tick only one:*

None 1 2 3 4 5 or more

Cannot recall Prefer not to say

Please provide details of misdiagnoses

……………………………………………………………………………………………………………………………………………………………………………………………………………………………………………………………………………………………………………………………………………………………………………………………………………………………………………………………………………………………………………………

Question 8

Please complete the table in the following way:

1. In the first column list select ALL of the aspects of your health affected by the rare condition.
2. In the second column please select ALL of the aspects of health affected by the rare condition for which you receive monitoring and/or treatment.

| Aspect of Health | Affected by rare condition  (tick ALL) | Receive monitoring and/or treatment  (tick ALL) |
| --- | --- | --- |
| Heart, circulatory (Cardiology) |  |  |
| Breathing, lungs (Respiratory Medicine) |  |  |
| Diabetes, hormones (Endocrinology) |  |  |
| Brain, nerves, spinal cord (Neurology) |  |  |
| Muscle, ligaments, joints (Rheumatology) |  |  |
| Bones, joints (Orthopaedics) |  |  |
| Skin (Dermatology) |  |  |
| Digestion (Gastroenterology) |  |  |
| Kidneys (Nephrology) |  |  |
| Mental Health (Psychiatry) |  |  |
| Chronic pain |  |  |
| Vision |  |  |
| Hearing |  |  |
| Behavioural difficulties |  |  |
| Learning difficulties |  |  |
| None of the above |  |  |
| Other: please add |  |  |
| Other: please add |  |  |

Question 9

Select from the list the health care professionals currently involved in care related to your rare condition.

*Tick ALL that apply*

GP

Hospital doctor

Community paediatrician

Specialist nurse

Practice or community nurse

Care coordinator

Health visitor

Genetic counsellor

Physiotherapist

Psychologist

Dietician

Speech and language therapist

Occupational therapist

Palliative Care specialist

Other, *provide details below*

……………………………………………………………………………………………………………………………………………………………………………………………………………………………………………………………………………………………………………………………………………………………………………………………………………………………………………………………………………………………………………………

Question 10

Do you receive regular treatment for any other health needs not related to your rare condition?

*Tick only one:*

Yes *provide details below*

No

Unsure

……………………………………………………………………………………………………………………………………………………………………………………………………………………………………………………………………………………………………………………………………………………………………………………………………………………………………………………………………………………………………………………

**Comment**

Is there any other information you would like to provide specifically relating to the rare condition or diagnosis?

Further information specific to the condition or diagnosis ………………………………………………………………………………………………………………………………………………………………………………………………………………………………………………………………………………………………………………………………………………………………………………………………………………………………………………………………………………………………………………………………………………………………………………………………………………………………………………………………………………………………………………………………………………………………………………………………………………………………………………………………………………………………………………………………………………………………………………………………………………………………………………………………………………………………………………………………………………………………………………………………………………………………………………………………………………………………………………………………………………………………………………………………………………………………………………………………………………………………………………………………………………………………………………………………………………………………………………………………………………………………………………………………………………………………………………………………………………………………………………………………………………………………………………………………………………………………………………………………………………………………………………………………………………………………………………………………………………………………………………………………………………………………………………………………………………………………………………………………………………………………………………………………………………………………………………………………………………………………………………………………………………………………………………………………………………………………………………………………………………………………………………………………………………………………………………………………………………………………………………………………………………………………………………………………………………………………………………………………………………………………………………………………

# Section B – Care coordinators

In this section we would like you to tell us about who is involved in care coordination.

*NOTE: A* ***formal care coordinator*** *is a professional with a recognised role in helping patients and carers manage a range of needs between different professionals or across care settings. They may be a full-time coordinator or may coordinate care as part of their main role, such as a GP.*

**Question 1**

Do you a have a formal care coordinator?

*Tick only one:*

Yes

No *Go to question 5*

Unsure *Go to question 5*

**Question 2**

Is the formal care coordinator employed specifically for the role, or do they coordinate care as part of another role (e.g., GP, specialist nurse)?

*Tick only one:*

Yes, the care coordinator is employed specifically for this role. *Go to question 4*

No, the care coordinator has another role.

Unsure *Go to question 4*

**Question 3**

What is the formal care coordinator’s main role?

*Tick only one:*

GP

Hospital doctors

Community paediatrician

Charity or patient support group representative

Specialist nurse

Practice or community nurse

Health visitor

Genetic counsellor

Physiotherapist

Occupational therapist

Palliative Care specialist

Other, *provide details below*

……………………………………………………………………………………………………………………………………………………………………………………………………………………………………………………………………………………………………………………………………………………………………………………………………………………………………………………………………………………………………………………

**Question 4**

Select the items on the list that are managed by the formal care coordinator.

*NOTE:* ***Care transitions*** *are permanent changes in care that can occur because of a change in needs, such as transfer from child to adult services, or a change in circumstances such as relocation from one region to another*

*Tick ALL that apply:*

Scheduling appointments

Liaising between health professionals

Liaising between health and non-health professionals (e.g., social worker, homecare)

Updating care plan

Ensuring availability of health records at appointments

Coordinating transitions of care

Advocating on your behalf

Liaising with patient to coordinate multi-disciplinary clinics

Out of hours contact

Contact for emergency or acute episodes

Arranging respite care

Other, *provide details below*

……………………………………………………………………………………………………………………………………………………………………………………………………………………………………………………………………………………………………………………………………………………………………………………………………………………………………………………………………………………………………………………

**Question 5**

In your opinion should a formal care coordinator be a single individual with responsibility for coordinating care?

*Tick only one:*

Yes

No

Unsure

**Question 6**

Do you consider yourself to be the main care coordinator regardless of whether you have a formal care coordinator?

*Tick only one:*

Yes

No

Unsure

**Question 7**

Select the items on the list where you feel you, or your carer, are the main care coordinator.

*Tick ALL that apply:*

Scheduling appointments

Liaising between health professionals

Liaising between health and non-health professionals

Updating care plan

Ensuring availability of health records at appointments

Coordinating transitions of care

Liaising with patient to coordinate multi-disciplinary clinics

Arranging respite care

Other, provide details below

……………………………………………………………………………………………………………………………………………………………………………………………………………………………………………………………………………………………………………………………………………………………………………………………………………………………………………………………………………………………………………………

**Question 8**

In this table we would like you to indicate whether you would prefer the aspect of care to be coordinated by you/your carer, or by a formal care coordinator.

| Aspect of care | Better coordinated by patient or carer  (tick box) | Better coordinated by formal care coordinator (tick box) |
| --- | --- | --- |
| Scheduling appointments |  |  |
| Liaising between health professionals |  |  |
| Liaising between health and non-health professionals (e.g., social worker, homecare) |  |  |
| Updating care plan |  |  |
| Ensuring availability of health records at appointments |  |  |
| Coordinating transitions of care |  |  |
| Liaising with patient to coordinate multi-disciplinary clinics |  |  |
| Arranging respite care |  |  |
| Other: please add |  |  |
| Other: please add |  |  |

**Question 9**

Select from the list the care transitions you have experienced.

*NOTE:* ***Care transitions*** *are permanent changes in care that can occur because of a change in needs such as transfer from child to adult services, or a change in circumstances such as relocation from one region to another.*

*Tick ALL that apply:*

Transition between child and adult services

Referrals between hospital doctors

Transition from GP to specialist care

Admission to a longer-term residential care facility

Transition to palliative care

No transitions in care *Go to Question 11*

Other, *provide details below*

……………………………………………………………………………………………………………………………………………………………………………………………………………………………………………………………………………………………………………………………………………………………………………………………………………………………………………………………………………………………………………………

**Question 10**

Have you had experience of a care transition which was managed by a formal care coordinator?

*Tick only one:*

- Yes, *provide details below*:
- No
- Unsure

……………………………………………………………………………………………………………………………………………………………………………………………………………………………………………………………………………………………………………………………………………………………………………………………………………………………………………………………………………………………………………………

**Question 11**

Regardless of your experience of these factors, please indicate if you agree or disagree with the statement in the table.

|  | Strongly agree | Agree | Neither agree or disagree | Disagree | Strongly disagree |
| --- | --- | --- | --- | --- | --- |
| Having a formal care coordinator improves the quality of care during transitions. |  |  |  |  |  |

Please tell us the reason for your answer:

……………………………………………………………………………………………………………………………………………………………………………………………………………………………………………………………………………………………………………………………………………………………………………………………………………………………………………………

**Question 12**

Select from the list your preferred option for who would be the formal care coordinator.

*Tick only one:*

Someone who is employed specifically as a care coordinator

Shared responsibility across professionals with no single coordinator

GP

Hospital doctor

Community paediatrician

Representative of patient group or charity

Specialist nurse

Practice or community nurse

Health visitor

Genetic counsellor

Physiotherapist

Occupational therapist

Palliative Care specialist

Other, *provide details below*

……………………………………………………………………………………………………………………………………………………………………………………………………………………………………………………………………………………………………………………………………………………………………………………………………………………………………………………………………………………………………………………

**Comment**

Is there any other information you would like to provide specifically relating to care coordinators?

Further information specific to care coordinators

………………………………………………………………………………………………………………………………………………………………………………………………………………………………………………………………………………………………………………………………………………………………………………………………………………………………………………………………………………………………………………………………………………………………………………………………………………………………………………………………………………………………………………………………………………………………………………………………………………………………………………………………………………………………………………………………………………………………………………………………………………………………………………………………………………………………………………………………………………………………………………………………………………………………………………………………………………………………………………………………………………………………………………………………………………………………………………………………………………………………………………………………………………………………………………………………………………………………………………………………………………………………………………………………………………………………………………………………………………………………………………………………………………………………………………………………………………………………………………………………………………………………………………………………………………………………………………………………………………………………………………………………………………………………………………………………………………………………………………………………………………………………………………………………………………………………………………………………………………………………………………………………………………………..

# Section C – Care Plans

In this section we would like you to tell us about your experience of using personalised, documented plans relating to your care (we use the term care plan).

*NOTE: A* ***care plan*** *is a paper or electronic document which describes the health services and support that are needed and should be agreed between patients, carers and professionals. The care plan may be a single document or it may be part of another record which includes non-health services such as an Educational Care and Health Plan (ECHP).*

**Question 1**

Do you have a care plan relating to your rare condition?

*Tick only one:*

Yes

No *Go to question 6*

Unsure *Go to question 6*

**Question 2**

Who is primarily responsible for keeping the care plan up to date?

*Tick only one:*

You, the patient

Your carer

Formal care coordinator

Shared responsibility between professionals

No one holds responsibility

GP

Hospital doctor

Specialist nurse

Representative of patient group or charity

Practice or community nurse

Health visitor

Genetic counsellor

Community paediatrician

Physiotherapist

Other, provide details below

……………………………………………………………………………………………………………………………………………………………………………………………………………………………………………………………………………………………………………………………………………………………………………………………………………………………………………………………………………………………………………………

**Question 3**

Were you involved in developing the care plan for your needs?

*Tick only one:*

Yes

No

Unsure

**Question 4**

Does the care plan adequately represent your needs?

*Tick only one:*

Yes

No

Unsure

**Question 5**

Select the aspects of care from the list that are addressed in the care plan.

*Tick ALL that apply*

General information and a medical summary

An assessment of current health needs

An assessment of current non-health needs (e.g., social care)

Documented health goals

Transition planning for changes in care

Scheduled reviews of the care plan

Plan of care for emergency or acute episodes

Out of office (non-urgent) contacts

Other, *provide details below:*

………………………………………………………………………………………………………………………………………………………………………………………………………………………………………………………………………………………………………………………………………………………………………………………………………………………………………………………………………………………………………………

**Question 6**

Select the 3 most useful items that should be included in a care plan.

*Pleae select 3 boxes:*

General information and a medical summary

An assessment of current health needs

An assessment of current non-health needs (e.g., social care)

Documented health goals

Transition planning for changes in care

Scheduled reviews of the care plan

Plan of care for emergency or acute episodes

Out of office hours (non-urgent) contacts

**Comment**

Is there any other information you would like to provide that is specific to care plans?

Further information specific to care plans

…………………………………………………………………………………………………………………………………………………………………………………………………………………………………………………………………………………………………………………………………………………………………………………………………………………………………………………………………………………………………………………………………………………………………………………………………………………………………………………………………………………………………………………………………………………………………………………………………………………………………………………………………………………………………………………………………………………………………………………………………………………………………………………………………………………………………………………………………………………………………………………………………………………………………………………………………………………………………………………………………………………………………………………………………………………………………………………………………………………………………………………………………………………………………………………………………………………………………………………………………………………………………………………………………………………………………………………………………………………………………………………………………………………………………………………………………………………………………………………………………………………………………………………………………………………………………………………………………………………………………………………………………………………………………………………………………………………………………………………………………………………………………………………………………………………………………………………………………………………………………………………………………………………………………………………………………………………………………………………………………………………………………………………………………………………………………………………………………………………………………………………………………………………………………………………………………………………………………………………………………………………………………………………………………………………………………………………………………………………………………………………………………………………………………………………………………………………………………………………………………………………………

# Section D – Specialist Centres

In this section we would like you to tell us about your experience of specialist centres.

*A* ***specialist centre*** *is a centralised facility that enables patients to see a number of health professionals in one visit. Usually, the professionals at specialist centres will be experts in rare and undiagnosed conditions. Non-health professionals may also see you at the same centre.*

**Question 1**

Is there a specialist centre available for you?

*Tick only one:*

Yes

No *Go to Section E*

Unsure *Go to Section E*

**Question 2**

Do you attend a specialist centre?

*Tick only one:*

Yes

No *provide details below, then go to Section E*

Unsure *Go to Section E*

If No, please tell us why you do not use the specialist centre:

Reason?………………………………………………………………………………………………………………………………………………………………………………………………………………………………………………………………………………………………………………………………………………………………………………………………………………………………………………………………………………………………………………………………………………………..…

**Question 3**

From the list select the health professionals that you see at the specialist centre.

*Tick ALL that apply:*

Doctors who are expert in rare or undiagnosed conditions

Doctors who are expert in aspects of health affected (e.g. neurologist)

Specialist nurse

Psychologist

Care coordinator

Genetic counsellor

Community paediatrician

Physiotherapist

Dietician

Speech and language therapist

Behavioral therapist

Occupational therapist

Other, provide details below:

…………………………………………………………………………………………………………………………………………………………………………………………………………………………………………………………………………………………………………………………………………………………………………………………………………………………………………………………………………………………………………………

**Question 4**

From the list select the services provided by the specialist centre.

*Tick ALL that apply:*

Appointments with an expert in rare conditions

Appointments to see different types of health professionals at the centre

Appointments to see non-health professionals (e.g., social worker)

Multiple appointments during a single visit

Appointments which are not in-person (e.g., virtual or telephone appointments)

Extended hours for appointments

Non-urgent, out-of-hours contact

Contact for acute or emergency episodes

Access to patient support groups or charities

Diagnostic and screening procedures

Support with routine admissions

Support during emergency admissions

Access to research opportunities

Other, please provide details below:

……………………………………………………………………………………………………………………………………………………………………………………………………………………………………………………………………………………………………………………………………………………………………………………………………………………………………………………………………………………………………………………

**Question 5**

From the list select 3 services of specialist centres which are, or would be, most helpful.

*Please select 3 boxes:*

Appointments with an expert in rare conditions

Appointments to see different types of health professionals at the centre

Appointments to see non-health professionals (e.g., social worker)

Multiple appointments during a single visit

Appointments which are not in-person (e.g., virtual or telephone appointments)

Extended hours for appointments

Non-urgent, out-of-hours contact

Contact for acute or emergency episodes

Access to patient support groups or charities

Diagnostic and screening procedures

Support with routine admissions

Support during emergency admissions

Access to research opportunities

Appointments with doctors who are experts in aspects of health affected (e.g. neurologists)

**Question 6**

Are there services you feel are missing from the care provided by the specialist centre?

*Tick only one:*

Yes, *provide details below:*

No

Unsure

**Question 7**

……………………………………………………………………………………………………………………………………………………………………………………………………………………………………………………………………………………………………………………………………………………………………………………………………………………………………………………………………………………………………………………

On average, how long is the time between appointments at the specialist centre?

*Tick only one:*

Less than 6 weeks

6 weeks to 3 months

4 to 6 months

7 to 12 months

13 to 18 months

More than 18 months

Unsure

**Question 8**

Please indicate if this time between appointments at the specialist centre is:

*Tick only one:*

Appropriate for needs

Too long – appointments are not frequent enough to meet needs

Too short – appointments are more frequent than needed

Unsure

**Comment**

Is there any other information you would like to provide that is specific to specialist centres?

Further information specific to specialist centres

…………………………………………………………………………………………………………………………………………………………………………………………………………………………………………………………………………………………………………………………………………………………………………………………………………………………………………………………………………………………………………………………………………………………………………………………………………………………………………………………………………………………………………………………………………………………………………………………………………………………………………………………………………………………………………………………………………………………………………………………………………………………………………………………………………………………………………………………………………………………………………………………………………………………………………………………………………………………………………………………………………………………………………………………………………………………………………………………………………………………………………………………………………………………………………………………………………………………………………………………………………………………………………………………………………………………………………………………………………………………………………………………………………………………………………………………………………………………………………………………………………………………………………………………………………………………………………………………………………………………………………………………………………………………………………………………………………………………………………………………………………………………………………………………………………………………………………………………………………………………………………………………………………………………………………………………………………………………………………………………………………………………………………………………………………………………………………………………………………………………………………………………………………………………………………………………………………………………………………………………………………………………………………………………………………………………………………………………………………………………………………………………………………………………………………………………………………………………………………………………………………………………

# Section E – Use of Health Services

In this section we would like to find out about your use of health services relating to your rare condition.

*This note will populate if attends specialist centre D2= YES Please record the combined total number of contacts you have had across ALL services over the past 12 months in the first column and then, tell us how many of that total were in a specialist centre in the second column.*

*This note will populate if attends specialist centre D2= NO/UNSURE Please record the total*

*number of contacts you have had across ALL services combined over the past 12 months.*

| SERVICE CONTACTS | TOTAL for past 12 months | #  SPECIALIST CENTRE?  **(if YES)** |
| --- | --- | --- |
| Outpatient appointment with expert in your condition or undiagnosed conditions |  |  |
| Outpatient appointment with doctors who are expert in the aspects of health affected (e.g. neurologist) |  |  |
| Outpatient appointment with specialist nurse |  |  |
| Outpatient appointment with physiotherapist |  |  |
| Outpatient appointment with care coordinator |  |  |
| Outpatient appointment with behavioral therapist |  |  |
| Outpatient appointment with psychologist |  |  |
| Outpatient appointment with occupational therapist |  |  |
| Outpatient appointment with genetic counsellor |  |  |
| Hospital outpatient appointments with other health professionals (provide the total number) |  |  |
| Visits to GP practice |  |  |
| Visits to practice nurse |  |  |
| GP home visit |  |  |
| Home visit from other health professional |  |  |
| Called 999 |  |  |
| Attended Emergency Department |  |  |
| Called NHS 111 |  |  |
| Visited an urgent care centre (including walk-in centre or minor injuries unit) |  |  |
| Attended acute admissions ward |  |  |
| Planned admission to hospital |  |  |
| Emergency admission to hospital |  |  |
| Appointment for diagnostic and screening procedures (including blood tests and routine scans) |  |  |
| Other NHS contacts (please detail which below ) |  |  |
| Contact with patient support groups/charities |  |  |

Please document any other contact you believe to be important.

Other NHS Services utilised in the past 12 months …………………………………………………………………………………………………………………………………………………………………………………………………………………………………………………………………………………………………………………………………………………………………………………………………………………………………………………………………………………………………………………………………………………………………………………………………………………………………………………………………………………………………………………………………………………………………………………………………………………………………………………………………………………………………………………………………………………………………………………………………………………………………………………………………………………………………………………………………………………………………………………………………………………………………………………………………………………………………………………………………………………………………………………………………………

# Section F (a) - costs of attending Specialist Centres

*Section will populate if = YES – you attend a specialist centre*

In this section we would like you to tell us more about the time and money costs involved with your visits to the specialist centre. Costs of care beyond specialist centres will be covered later.

**Question 1**

How far away is the specialist centre from your home?

Miles

**Question 2**

From leaving home to returning home, how long on average does it take to attend the specialist centre?

mins

Hours

**Question 3**

Which mode of transportation do you mostly use to travel to your specialist centre?

*Tick only one:*

- Car/Motorbike
- Bus
- Overground train
- Underground train
- Bike
- Taxi / private hire
- Hospital transport
- Walk
- Other, *provide detail below*

…………………………………………………………………………………………………………………………………………………………………………………………………………………………………………………………………………………………………………………………………………………………………………………………………………………………………………………………………………………………………………………

**Question 4**

How many adults and children normally attend visits to the specialist centre (and are included in the costs above)?

Adults

Children

**Question 5**

What is the average total cost of travel for everyone who usually attends the specialist centre appointments?

(Please provide total costs of transport only for a return journey to the nearest £10)

*NOTE: Car fuel costs on average £1 for every 10 miles.*

£

**Question 6**

On average, what is the total cost per visit of other expenses you pay for one appointment at the specialist centre (e.g., childcare, parking, food and drink)?

£

**Question 7**

Are you able to reclaim the expenses you incur for attending the specialist centre?

Yes

No

Unsure

**Question 8**

If you are working, how many days in the average year do you have to take off work to attend the health care appointments at the specialist centre?

Days off/12months

N/A not working

**Comment**

Is there any other information you would like to provide that is specific to the time and cost of attending specialist centres?

Information specific to costs and time of accessing care ……………………………………………………………………………………………………………………………………………………………………………………………………………………………………………………………………………………………………………………………………………………………………………………………………………………………………………………………………………………………………………………………………………………………………………………………………………………………………………………………………………………………………………………………………………………………………………………………………………………………………………………………………………………………………………………………………………………………………………………………………………………………………………………………………………………………………………………………………………………………………………………………………………………………………………………………………………………………………………………………………………………………………………………………………………………………………………………………………………………………………………………………………………………………………………………………………………………………………………………………………………………………………………………………………………………………………………………………………………………………………………………………………………………………………………………………………………………………………………………………………………………………………………………………………………………………………………………………………………………………………………………………………………………………………………………………………………………………………………………………………………………………………………………………………………………………………………………………………………………………………………………………………………………………………………………………………………………………………………………………………………………………………………………………………………………………………………………………………………………………………………………………………………………………………………………………………………………………………………………………………………………………………………………………………………………………………………………………………………………………………………………………………………………………………………………………………………………………………………………

# Section F (b) - Costs of accessing health care services

*NOTE will populate if attends specialist centre D2= NO* In this section we would like you to tell us about the time involved and the costs associated with accessing health services.

*NOTE will populate if attends specialist centre D2= YES* In this section we would like you to tell us about the time involved and the cost associated with accessing health services related to the rare condition of the person you care for that are not provided by the specialist centre, for example, costs incurred when attending GP appointments.

**Question 1**

Over the last 12-months how many different hospitals were involved in care relating to the rare condition?

# hospitals

**Question 2**

From the time you leave home to returning home how long, on average, is the journey to the hospital attended most often?

hours

mins

**Question 3**

Which mode of transportation is predominately used to travel to hospital visits?

*Tick only one:*

Car/Motorbike

Bus

Overground train

Underground train

Bike

Taxi / Private hire

Hospital transport

Walk

Other, *provide detail below*

…………………………………………………………………………………………………………………………………………………………………………………………………………………………………………………………………………………………………………………………………………………………………………………………………………………………………………………………………………………………………………………

**Question 4**

How many adults and children normally attend visits to the hospital (and are included in the costs above)?

Adults

Children

**Question 5**

What is the average total cost of travel for everyone who usually attends the hospital appointments?

(Please provide total costs of transport only for a return journey to the nearest £10)

*NOTE: Car fuel costs on average £1 for every 10 miles.*

£

**Question 6**

On average, what is the total cost per visit of other expenses you pay for one hospital appointment (e.g., childcare, parking, food and drink)?

£

**Question 7**

On average how many hours per month do you spend dealing with the organisation of health care services such as arranging appointments, managing prescriptions etc.?

hours per month

**Question 8**

If you are working, how many days in the average year do you have to take off work to attend appointments related to your rare condition?

Days off/12months

N/A not working

**Question 9**

Has your rare condition affected your ability to work?

*Tick only one:*

No, I am still in education

No, there has been no change in my working pattern due to my condition

No, I have retired as planned

Yes, I have never worked because of my condition

Yes, I have paused or stopped my education

Yes, I have taken early retirement or left work because of my condition

Yes, I have changed jobs because of my condition

Yes, I have changed to working fewer hours because of my condition

Other *Please specify below*

Prefer not to say

…………………………………………………………………………………………………………………………………………………………………………………………………………………………………………………………………………………………………………………………………………………………………………………………………………………………………………………………………………………………………………………

**Comment**

Is there any other information you would like to provide that is specific to the time and cost of accessing health care?

Information specific to costs and time of accessing care ……………………………………………………………………………………………………………………………………………………………………………………………………………………………………………………………………………………………………………………………………………………………………………………………………………………………………………………………………………………………………………………………………………………………………………………………………………………………………………………………………………………………………………………………………………………………………………………………………………………………………………………………………………………………………………………………………………………………………………………………………………………………………………………………………………………………………………………

# Section G - Quality of care impacts

In this section we would like you to indicate how much you agree or disagree with the statements in each table regardless of your prior knowledge or experience of these factors RANDOMISE

| **Statement 1** | Strongly agree | Agree | Neither agree or disagree | Disagree | Strongly disagree |
| --- | --- | --- | --- | --- | --- |
| Having a care plan improves the quality of care for people with rare conditions |  |  |  |  |  |

| **Statement 2** | Strongly agree | Agree | Neither agree or disagree | Disagree | Strongly disagree |
| --- | --- | --- | --- | --- | --- |
| Having a care coordinator improves the quality of care for people with rare conditions |  |  |  |  |  |

| **Statement 3** | Strongly agree | Agree | Neither agree or disagree | Disagree | Strongly disagree |
| --- | --- | --- | --- | --- | --- |
| Attending a specialist centre improves the quality of care for people with rare conditions |  |  |  |  |  |

**Comment**

If you would like to explain any of your answers to the tables in Section G further, please do so in the box below:

……………………………………………………………………………………………………………………………………………………………………………………………………………………………………………………………………………………………………………………………………………………………………………………………………………………………………………………………………………………………………………………………………………………………………………………………………………………………………………………………………………………………………………………………………………………………………………………………………………………………………………………………………………………………………………………………………………………………………………………………………………………………………………………………………………………………………………………………………………………………………………………………………………………………………………………………………………………………………………………………………………………………………………………………………………………………………………………………

# Section H – Discrete Choice Experiment (DCE)

In the following section you will see two different scenarios. We would like you to tell us which one you would prefer.

You will be asked to do this six times, each time with a slightly different set of options, so please make sure you read each scenario carefully before ticking the option you prefer (Service A or Service B).

Each scenario will have different characteristics for six aspects of care coordination; we have listed them all below so you know what they are before you start.

| **Cost of attending all appointments over one year** | | | | | |
| --- | --- | --- | --- | --- | --- |
| Describes the cost to patients and carers of attending all health care appointments over one year (including travel costs, time off work, childcare costs, subsistence). | | | | | |
| Possible values | | | | | |
| £200 | £400 | | £1000 | | £2000 |
|  | | | | | |
| **Access to health records** | | | | | |
| Describes the way in which health records are shared by different health professionals in the same centre or across different health settings. | | | | | |
| Possible values | | | | | |
| Health records are not shared. Test results and clinic letters are sent through the post. | | | Electronic health records are immediately accessible to staff. | | |
|  | | | | | |
| **Clinical expertise** | | | | | |
| The type of medical professional who is the lead consultant and makes the majority of decisions regarding medical care. | | | | | |
| Possible values | | | | | |
| The lead consultant is a medical expert in your specific condition | | | The lead consultant is a medical expert in the area of the body primarily affected byyour condition (e.g., neurologist) | | |
|  | | | | | |
| **Role of care coordinator** | | | | | |
| Describes the amount of involvement of a formal care coordinator who is a health care professional. | | | | | |
| Possible values | | | | | |
| Care is provided without the support of a care coordinator | | Care is entirely coordinated on your behalf by a care coordinator | | You have a named care coordinator and you decide how they support you | |
|  | | | | | |
| **Access to Specialist Centre** | | | | | |
| A Specialist Centre enables patients to see a number of health professionals in one visit. Generally, they will be experts in rare and undiagnosed conditions. Non-health professionals may also see patients at the same centre*.* | | | | | |
| Possible values | | | | | |
| You **do not** have access to a specialist centre | | | A specialist centre is available | | |
|  | | | | | |
| **Documented emergency Plan** | | | | | |
| A formal emergency plan describes the correct treatment which should be provided in urgent situations and contact details for a health professional who has knowledge of the specific condition. | | | | | |
| Possible values | | | | | |
| There is a documented emergency plan in place | | | No documented emergency plan exists | | |

**First an example...**A person has been asked to consider the characteristics of Service A and Service B listed below and then tick the box to indicate which of the two services s/he would choose.

|  | Service A | Service B |
| --- | --- | --- |
| Cost of attending all appointments over one year | £200 | £1,000 |
| Access to health records | Health records are not shared. Test results and clinic letters are sent through the post. | Electronic health records are immediately accessible to staff. |
| Clinical expertise | The lead consultant is a medical expert in your specific condition | The lead consultant is a medical expert in the area of the body primarily affected by your condition (e.g., neurologist) |
| Role of coordinator | Care is provided without the support of a care coordinator | Care is entirely coordinated on your behalf by a care coordinator |
| Access to specialist centre | A specialist centre is available | You do not have access to a specialist centre |
| Documented emergency plan | There is a documented emergency plan in place | No documented emergency plan exists |

If, on balance, the person would prefer Service B as described rather than Service A, s/he would have ticked the box for Service B:

Service A  Service B

# Scenario 1

|  | **Service A** | **Service B** |
| --- | --- | --- |
| **Cost of attending all appointments over one year** | £200 | £1,000 |
| **Access to health records** | Health records are not shared. Test results and clinic letters are sent through the post. | Electronic health records are immediately accessible to staff. |
| **Clinical expertise** | The lead consultant is a medical expert in your specific condition | The lead consultant is a medical expert in the area of the body primarily affected by your condition |
| **Role of coordinator** | Care is provided without the support of a care coordinator | You have a named care coordinator and you decide how they support you |
| **Access to specialist centre** | You **do not** have access to a specialist centre | A specialist centre is available |
| **Documented emergency plan** | There is a documented emergency plan in place | No documented emergency plan exists |

Which Service would you choose? (Tick one box only)

Service A Service B

# Scenario 2

|  | **Service A** | **Service B** |
| --- | --- | --- |
| **Cost of attending all appointments over one year** | £1,000 | £400 |
| **Access to health records** | Electronic health records are immediately accessible to staff. | Health records are not shared. Test results and clinic letters are sent through the post. |
| **Clinical expertise** | The lead consultant is a medical expert in your specific condition | The lead consultant is a medical expert in the area of the body primarily affected by your condition |
| **Role of coordinator** | You have a named care coordinator and you decide how they support you | Care is provided without the support of a care coordinator |
| **Access to specialist centre** | You **do not** have access to a specialist centre | A specialist centre is available |
| **Documented emergency plan** | No documented emergency plan exists | There is a documented emergency plan in place |

Which Service would you choose? (Tick one box only)

Service A Service B

# Scenario 3

|  | **Service A** | **Service B** |
| --- | --- | --- |
| **Cost of attending all appointments over one year** | £1,000 | £400 |
| **Access to health records** | Electronic health records are immediately accessible to staff. | Health records are not shared. Test results and clinic letters are sent through the post. |
| **Clinical expertise** | The lead consultant is a medical expert in the area of the body primarily affected by your condition | The lead consultant is a medical expert in your specific condition |
| **Role of coordinator** | Care is entirely coordinated on your behalf by a care coordinator | You have a named care coordinator and you decide how they support you |
| **Access to specialist centre** | You **do not** have access to a specialist centre | A specialist centre is available |
| **Documented emergency plan** | There is a documented emergency plan in place | No documented emergency plan exists |

Which Service would you choose? (Tick one box only)

Service A Service B

# Scenario 4

|  | **Service A** | **Service B** |
| --- | --- | --- |
| **Cost of attending all appointments over one year** | £2,000 | £1,000 |
| **Access to health records** | Electronic health records are immediately accessible to staff. | Health records are not shared. Test results and clinic letters are sent through the post. |
| **Clinical expertise** | The lead consultant is a medical expert in your specific condition | The lead consultant is a medical expert in the area of the body primarily affected by your condition |
| **Role of coordinator** | Care is entirely coordinated on your behalf by a care coordinator | Care is provided without the support of a care coordinator |
| **Access to specialist centre** | A specialist centre is available | You **do not** have access to a specialist centre |
| **Documented emergency plan** | No documented emergency plan exists | There is a documented emergency plan in place |

Which Service would you choose? (Tick one box only)

Service A Service B

# Scenario 5

|  | **Service A** | **Service B** |
| --- | --- | --- |
| **Cost of attending all appointments over one year** | £200 | £2,000 |
| **Access to health records** | Electronic health records are immediately accessible to staff. | Health records are not shared. Test results and clinic letters are sent through the post. |
| **Clinical expertise** | The lead consultant is a medical expert in your specific condition | The lead consultant is a medical expert in the area of the body primarily affected by your condition |
| **Role of coordinator** | Care is provided without the support of a care coordinator | You have a named care coordinator and you decide how they support you |
| **Access to specialist centre** | A specialist centre is available | You **do not** have access to a specialist centre |
| **Documented emergency plan** | No documented emergency plan exists | There is a documented emergency plan in place |

Which Service would you choose? (Tick one box only)

Service A Service B

|  | **Service A** | **Service B** |
| --- | --- | --- |
| **Cost of attending all appointments over one year** | £1,000 | £200 |
| **Access to health records** | Electronic health records are immediately accessible to staff. | Health records are not shared.   Test results and clinic letters are sent through the post. |
| **Clinical expertise** | The lead consultant is a medical expert in your specific condition | The lead consultant is a medical expert in the area of the body primarily affected by your condition |
| **Role of coordinator** | Care is provided without the support of a care coordinator | You have a named care coordinator and you decide how they support you |
| **Access to specialist centre** | You **do not** have access to a specialist centre | A specialist centre is available |
| **Documented emergency plan** | There is a documented emergency plan in place | No documented emergency plan exists |

# Scenario 6

Which Service would you choose? (Tick one box only)

Service A Service B

Please tell us how easy or difficult you found this section (Section H – DCE) to complete.

| 🞏 | Very easy |
| --- | --- |
| 🞏 | Easy |
| 🞏 | Difficult |
| 🞏 | Very difficult |

If you found this section of the questionnaire difficult or very difficult to complete please tell us why in the box below.

Reason?…………………………………………………………………………………………………………………………………………………………………………………………………………………………………………………………………………………………………………………………………………………………………………………………………………………………………………………………………………………………………… …………………………………………………………………………………………………………………………………………………………………………………………………………………………………………………………………………………………………………………………………………………………………………………………………………………………………………………………………………………………………………………………………………………………………………………………………………………………………………………………………………………………………………………………………………………………………………………………………………………………………………………………………………

# Section I - Ranking

Please rank the different aspects of care coordination below in order of their importance to you with 1 being the aspect you consider most important for care coordination and 6 being the aspect you consider least important for care coordination.

| **ASPECTS OF COORDINATED CARE** | **DEFINITION** | RANK IN ORDER OF IMPORTANCE 1=most important  6= least important |
| --- | --- | --- |
| **Cost of attending all appointments over one year** | Describes the cost of attending all health care appointments over one year (including travel costs, time off work, childcare costs, subsistence). |  |
| **Access to health records** | Describes the way in which health records are shared by different health professionals in the same centre or across different settings. |  |
| **Clinical expertise** | The expertise of the doctor who is the lead consultant and makes the majority of decisions regarding medical care |  |
| **Role of care coordinator** | Describes the amount of involvement of a formal care coordinator who is a health care professional |  |
| **Access to specialist centre** | A specialist centre enables patients to see a number of health professionals in one visit. Generally, they will be experts in rare and undiagnosed conditions. Non-health professionals may also see patients at the same centre. |  |
| **Documented emergency plan** | A formal emergency plan describes the correct treatment health professionals should provide in urgent situations and contact details for a health professional who has knowledge of the specific condition. |  |

# Information about you

In this section we would like to collect some information about you.

**Question 1**

What is your age?

*Tick only one:*

18–24

25-34

35-44

45-54

55-64

65-74

75+

Prefer not to say

Question 2

What is your sex?

*Tick only one:*

🞎 Female 🞎 Male 🞎 Other 🞎 Prefer not to say

**Question 3**

Do you live alone?

*Tick only one:*

Yes, I live alone

No, I live with a spouse or partner

No, I live with family members or friends

No, I live with a carer

Prefer not to say

Question 4

Which geographical region of the UK do you live in?

*Tick only one:*

| East of England  East Midlands  London  North East & Cumbria  Northern Ireland  North West of England  Scotland | South East of England  South West of England  Wales  West Midlands  Yorkshire  Prefer not to say  Other [Please specify below] |
| --- | --- |

…………………………………………………………………………………………………………………………………………………………………………………………………………………………………………………………………………………………………………………………………………………………………………………………………………………………………………………………………………………………………………

**Question 6**

Which category best describes your ethnic group?

*Tick only one:*

White

Mixed or multiple ethnic groups

Indian

Pakistani

Bangladeshi

Chinese

Any other Asian background

Black, African, Black Caribbean, Black British

Prefer not to say

Other ethnic group, *provide detail below:*

Other…………………………………………………………………………………………………………………………………………………………………………………………………………………………………………………………………………………………………………………………………………………………………………………………………………………………………………………………………………………………………………………

**Question 7**

What is your highest level of educational qualification?

*Tick only one:*

No formal qualifications or equivalent

O level or GCSE, or equivalent

ONC or BTEC, or equivalent

A level (‘Higher’ in Scotland) or equivalent

Higher-education qualification below degree level or equivalent

Degree or higher degree or equivalent

Prefer not to say

**Comment**

Please use this box if you would like to tell us anything else about yourself in relation to any aspect of care coordination.

Comment…………………………………………………………………………………………………………………………………………………………………………………………………………………………………………………………………………………………………………………………………………………………………………………………………………………………………………………………………………………………………………………………………………………………

**Question 8:** Please tell us the name of the organisation which provided you with this survey**:**

**……………………………………………………………………………………………….**

Thank you so much, you made it ☺! **We hope you enjoyed taking part in this study. Your answers will be very valuable to us**. If you have any comments, questions or suggestions about this survey or the CONCORD study in general, please write them in the box below

Comment…………………………………………………………………………………………………………………………………………………………………………………………………………………………………………………………………………………………………………………………………………………………………………………………………………………………………………………………………………………………………………………………………………………………………………………………………………………………………………………………………………………………………………………………………………………………………………………………………………………………………………………………………………………………………………………………………………………………………………………………………………………………………………………………………………………………………………………………………………………………………………………………………………………………………………………………………………………………………………………………………………………………………………………………………………………………………………………………………………………………………………………………………………………………………………………………………………………………………………………………………………………………………………………………………………………………………………………………………………………………………………………………

If you have any questions about this survey please contact:

Emma Hudson email:e.hudson@ucl.ac.uk Free Telephone Number: 0800 084 2783.

To find out more about the CONCORD study or to access the results of the study when they become available, please go to: https://www.geneticalliance.org.uk/our-work/healthcare-and-delivery/coordinated-care-of-rare-diseases-concord/

**Thank you for taking part.**
