# Supplementary material for: Experiences of coordinated care for people in the UK affected by rare diseases: cross-sectional survey of patients, carers, and healthcare professionals
Source: Orphanet J Rare Dis. 2023 Nov 23;18:364. doi: 10.1186/s13023-023-02934-9 (PMC10668407; doi:10.1186/s13023-023-02934-9)
Supplement: Supplementary file 3 — Additional file 3. Copy of healthcare professional survey. [file 13023_2023_2934_MOESM3_ESM.docx]

**Participant Information Sheet for CONCORD Survey**

**Title of Study:** COORDINATED CARE OF RARE DISEASES (CONCORD)

**Information about our survey:**

Thank you for taking the time to look at our survey.

We are interested in learning about the health care experiences of people with rare conditions, specifically about how care is coordinated when people have a range of different needs.

This survey is part of a larger study, called Coordinated Care of Rare Diseases (CONCORD), which is sponsored by University College London (UCL), funded by the UK National Institute for Health Research (NIHR) and approved by an independent ethics committee:

Research Ethics Committee ID Number: 19/LO/0250.

**Name and Contact Details of the Researcher(s): 
Emma Hudson:** [**e.hudson@ucl.ac.uk**](mailto:e.hudson@ucl.ac.uk)

**Name and contact details of company providing survey:**

**Accent, Alison Lawrence: Alison.lawrence@accent-mr.com**

**Name and Contact Details of the Principal Researcher: 
Stephen Morris:**[sm2428@medschl.cam.ac.uk](mailto:sm2428@medschl.cam.ac.uk)

**What is the study about?**

The COordiNated Care of Rare Diseases (CONCORD) study aims to explore how care for rare diseases is coordinated in the UK and how patients, families and healthcare professionals would like them to be coordinated. You are being invited to take part in a survey, which is part of the overall CONCORD study. The findings will help us understand more about care coordination and preferences for care coordination. This study is being conducted by researchers in the Department of Applied Health Research at University College London.

**Can I participate?**

You can participate if:

- you are a patient with a rare, ultra-rare, or undiagnosed condition **or**
- you have experience of caring for a patient with a rare, ultra-rare, or undiagnosed condition **or**
- you are a health professional who has experience in caring for patients that fit this definition.

We are aiming to collect around 1500 responses.

**Do I have to take part?**

No. Taking part in this survey is voluntary.

**What would taking part involve?**

If you decide to take part, you will be asked to complete a survey about care coordination. The first part of the survey asks you to tell us about your experiences of care coordination and your preferences for how care should be coordinated. The second part of the survey will ask you to make a choice between two different scenarios of care coordination. This is a really helpful way of showing us what your priorities are for these different aspects of care coordination. The survey will be managed by a professional survey company who will have a service level agreement with UCL outlining data protections.

You can complete this survey online, or if you’d prefer, you can request to complete the survey in hardcopy, electronically, or over the phone. Requests for hardcopies, electronic copies, or completion over the phone can be made directly with the professional survey company, Accent, by emailing CONCORD@accent-mr.com or calling their FREEPHONE number 0800 084 2783).Your consent to participate in this survey will be implied if you submit an online, electronic, or hardcopy version of the survey. Your consent will be confirmed verbally if you choose to take the survey over the phone

**How long will it take to complete the survey?**

The survey takes most people up to 30 minutes to complete but if you are doing it online you can take a break at any point and finish it when you are ready by using the same link to the website.

**What are the possible disadvantages and risks of taking part?**

The survey will take some time to complete but there are no other foreseeable disadvantages or risks of taking part in this survey.

**What are the possible benefits of taking part?**

There are no immediate benefits for those people participating in the project, however, it is hoped that this study will support improvements in how care is coordinated for people with rare conditions across the NHS in England.

**What if something goes wrong?**

If you have any queries about the questionnaire, please contact Emma Hudson e.hudson@ucl.ac.uk. If you experience any technical difficulties with filling in the questionnaire, please call the survey company on FREEPHONE helpline number 0800 084 2783 or email CONCORD@accent-mr.com. If you have any complaints about this study, please contact the Principal Researcher, Prof Stephen Morris at [sm2428@medschl.cam.ac.uk](mailto:sm2428@medschl.cam.ac.uk). Should you feel your complaint has not been handled to your satisfaction, you can contact the UCL Research and Development office at [uclh.randd@nhs.net](mailto:uclh.randd@nhs.net).

UCL holds insurance against claims from participants for harm caused by their participation in this research. Participants may be able to claim compensation if they can prove that UCL has been negligent.

**Will my taking part in this project be kept confidential?**

We will not tell anyone that you have taken part in this survey. If you need an electronic copy or hardcopy of this survey, or decide to complete it over the phone with the survey company, the survey company will need to have your contact details, but these details will only be used for this purpose and will not be shared with anyone else. All information collected during the study will be kept strictly confidential. The results of the survey will be used in written reports, academic publications, conference publications or any other material produced for the study. We will not use your name and will make every effort to protect your identity.

**Limits to confidentiality**

Confidentiality will be respected subject to legal constraints and professional guidelines.

**How will my personal information be handled?**

The information you provide as part of the survey will be stored and handled by a professional survey distribution company with whom UCL has a service level agreement outlining data protections. The survey company will process your answers in confidence. They will then send your answers to researchers at UCL to be analysed. The survey company and UCL will store your responses in line with the Data Protection Act 1998 and new General Data Protection Regulation (GDPR). Any of your identifiable data we collect will be stored securely for up to three years after the end of the project and then destroyed securely. Anonymised data will be archived for 20 years. Your responses to the questionnaire will be processed only for the purposes required for this research project. No personal data will be transferred outside the European Economic Area (EEA). Data used in any publication will be fully anonymised.

If you are concerned about how your response data is being processed, please contact UCL in the first instance at data-protection@ucl.ac.uk. If you remain unsatisfied, you may wish to contact the Information Commissioner’s Office (ICO). Contact details, and details of data subject rights, are available on the ICO website at: https://ico.org.uk/for-organisations/data-protection-reform/overview-of-the-gdpr/individuals-rights/

**Data Protection Privacy Notice**By completing this questionnaire, you are giving your consent for your personal details and relevant health information to be held and analysed by a survey company and UCL for the purposes of this study only**.**

Please note:

- Your personal information will be handled securely and anonymised before analysis and before any publication.
- Your personal information will not be released by anyone working on the study unless required by law or where there is a clear overriding public interest.

University College London (UCL) is the sponsor for this study based in the United Kingdom. We will be using information from you in order to undertake this study and will act as the data controller for this study. This means that we are responsible for looking after your information and using it properly. UCL will keep identifiable information about you for three years after the study has finished.

Your rights to access, change or move your information are limited, as we need to manage your information in specific ways in order for the research to be reliable and accurate. To safeguard your rights, we will use the minimum personally-identifiable information possible. You can find out more about how we use your information by contacting the UCL Data Protection Office at [data-protection@ucl.ac.uk](mailto:data-protection@ucl.ac.uk) or by looking at the following website: <https://www.ucl.ac.uk/legal-services/privacy/participants-health-and-care-research-privacy-notice>

Your answers to the questionnaire will be processed for the purposes outlined in this notice. By clicking on the consent to participate button below you are agreeing that you have read and understood the information contained in this participant information sheet.

**START THE SURVEY**

# Completing the survey

Please answer each question by ticking the corresponding box or selecting options from the lists provided. For each question that asks you to state a preference please select the answer that best represents the choice you would make for the patients you care for (this may, or may not, be the one you think they themselves would choose).

For some questions you may tick more than one box. Sometimes you will find the box you have ticked moves you on to other questions so you do not spend time on questions that do not apply to you. If you make a mistake, simply change your selection to the correct answer. You can return to a previous question using the back arrow if you want to do so.

There are comment boxes throughout the survey if you would like to give us more information to support your answer. If you do not wish to do so please leave these boxes blank.

The last section of the survey looks a bit different. You will see a number of questions where there are two options to choose from and we would like you to tell us which one you prefer for the patients you care for. This is a really helpful way of showing us what your priorities are for these different aspects of care coordination.

Please do not put your name or address anywhere on the questionnaire.

Some of the terms we use in the survey might have different meanings to different people. We explain what we mean by these terms as they appear in the survey, but you can also find them all listed (here*)

Finally, thank you for participating in the survey, we really do appreciate the time you give up in order to share your experiences with us and value all of the information provided.

THE CONCORD RESEARCH TEAM

Summary of survey terms **will appear when link is activated*

**Rare condition: In Europe a condition is considered to be a rare condition when it affects fewer than 5 in 10,000 people.**

**Ultra rare condition**: A disease is generally considered to be ultra-rare if it affects one patient per 50,000 people (or, fewer than 20 patients per million of population).

An **undiagnosed condition** is a condition which health professionals determine to be rare but tests have not provided a diagnosis. The condition might be complex and might have a genetic cause.

**NOTE: Unless specified in the question, the term rare condition denotes rare, ultra-rare and undiagnosed conditions.**

NOTE: for the purposes of the survey we use the term condition to refer to all rare conditions, diseases and syndromes.

A **specialist centre** is a centralised facility that enables patients to see a number of health professionals in one visit. Usually, the professionals at specialist centres will be experts in rare and undiagnosed conditions. Non-health professionals may also see patients at the same centre.

A **formal care coordinator** is a professional with a recognised role in helping patients and carers manage a range of needs between different professionals or across care settings. They may be a full-time coordinator or may coordinate care as part of their main job, such as a GP.

A **care plan** is a paper or electronic document which describes the health services and support that is needed and should be agreed between patients, carers and professionals. The care plan may be a single document or it may be part of another record which includes non-health services such as an Educational Care and Health Plan (ECHP).

A **care transition** is a permanent change in care that can occur because of a change in needs, such as transfer from child to adult services, or circumstances such as relocation from one region to another.

# Suitability for survey

Please read the following statements carefully and tick the appropriate box.

If you answer NO for any of the statements, you are unfortunately not eligible to take part in the survey.

If you tick YES to all of the statements, you are eligible to take part in the survey.

| CRITERIA | YES | NO |
| --- | --- | --- |
| I have experience of caring for people with rare or undiagnosed conditions (can be past or current experience). |  |  |
| I am 18 years old or over. |  |  |
| I am living in the UK and was or am currently employed by the NHS. |  |  |
| I have read and understood the information regarding the survey. |  |  |
| I understand that by completing this questionnaire, I am giving consent for my personal details and relevant health information to be held and analysed by a survey company and UCL for the purposes of this study as outlined in the information provided. |  |  |
| I am aware of who I should contact if I wish to discuss any aspect of the study. |  |  |
| I give permission for the data I provide to be archived at University College London for up to three years after the end of the project. |  |  |

Did you find out about the survey from your NHS employer?

*Tick only one:*

Yes

No *Go to Section A*

Unsure *Go to Section A*

If Yes, please tell us the name of the hospital or health care facility.

Care provider …………………………………………………………………………………………………………………………………………………………………………………………………………………………………………………………………………………………………………………………………………………………………………………………………………………………………………………………………………………………………………………

# Section A – Experience of rare conditions

This section focuses on your role in caring for people with rare or undiagnosed conditions.

**Question 1**

Do you care for people with rare conditions?

*Tick only one:*

Yes

No

Question 2

Do you care for people with undiagnosed or suspected genetic conditions?

*Tick only one:*

Yes

No

DP LOGIC CHECK: if Q1 and Q2 both ‘no’ show following error message “To be eligible to complete this survey you need to be caring for people with rare, undiagnosed or suspected genetic conditions. Please review your answer to these questions”. Give option to return to Q1 or close.

Question 3

What part of care delivery for people with rare conditions are you mainly involved in?

*Please tick ALL that apply*

Diagnosing condition

Providing information/signposting, or counselling

Long-term care planning following diagnosis

Long-term care planning in the absence of a diagnosis

Other, *provide detail below*

Question 4

……………………………………………………………………………………………………………………………………………………………………………………………………………………………………………………………………………………………………………………………………………………………………………………………………………………………………………………………………………………………………………………

Are you an expert with training in specific types of rare conditions?

*Tick only one:*

Yes, *list the conditions below*

No

……………………………………………………………………………………………………………………………………………………………………………………………………………………………………………………………………………………………………………………………………………………………………………………………………………………………………………………………………………………………………………………

**Question 5**

In your experience, if diagnosis is possible, what is the average length of time from a patient’s first contact with a health professional regarding symptoms, to diagnosis?

________months if known

_________years

Do not know Prefer not to say

# Section B – Care coordinators

This section aims to understand the role of formal care coordinators in supporting people with rare conditions.

*NOTE:*  *A* ***formal care coordinator*** *is a professional with a recognised role in helping patients and carers manage a range of needs between different professionals or across care settings. They may be a full-time coordinator or may coordinate care as part of their main role, such as a GP.*

**Question 1**

Do the majority of your patients have a formal care coordinator?

*Tick only one:*

Yes

No *Go to question 7*

Unsure *Go to question 7*

**Question 2**

Are you a formal care coordinator?

*Tick only one:*

Yes

No

**Question 3**

For patients with rare conditions, are the majority of formal care coordinators employed specifically for the role, or do they coordinate care as part of another role (e.g., GP, specialist nurse)?

*Tick only one*:

Yes, the majority are employed specifically as care coordinators. *Go to question 5*

No, usually, care coordination is undertaken as part of another role.

Unsure *Go to question 5*

**Question 4**

Please indicate who, in the majority of cases, is fulfilling the role of formal care coordinator.

*Tick only one:*

GP

Hospital doctor

Community paediatrician

Charity or patient support group representative

Specialist nurse

Practice or community nurse

Health visitor

Genetic counsellor

Physiotherapist

Occupational therapist

Palliative Care specialist

Other, *provide details below*

……………………………………………………………………………………………………………………………………………………………………………………………………………………………………………………………………………………………………………………………………………………………………………………………………………………………………………………………………………………………………………………

**Question 5**

Do you think a care coordinator should always be a single individual with responsibility for this activity?

*Tick only one:*

Yes

No *Provide the reason for your answer*

Unsure *Provide the reason for your answer*

……………………………………………………………………………………………………………………………………………………………………………………………………………………………………………………………………………………………………………………………………………………………………………………………………………………………………………………………………………………………………………………

**Question 6**

In your experience which of the following items are organised by a formal care coordinator for your patients?

*Tick ALL that apply:*

Scheduling appointments

Liaising between health professionals

Liaising between health and non-health professionals (e.g. social worker, homecare)

Updating care plan

Ensuring availability of up-to-date health records at appointments

Coordinating transitions of care

Acting as an advocate for the patient

Liaising with patient to coordinate multi-disciplinary clinics

Out of hours contact

Contact for emergency or acute episodes

Arranging respite care

Other, *provide details below*

……………………………………………………………………………………………………………………………………………………………………………………………………………………………………………………………………………………………………………………………………………………………………………………………………………………………………………………………………………………………………………………

**Question 7**

What are the main factors that determine whether someone with a rare condition will have access to a formal care coordinator?

*Tick ALL that apply*

Complexity of disease

Request of patient / carer / family

Extent of patient’s need for support

Patient’s existing support system (number and role of carers)

Distance from specialist centre

Caseload of health care professionals involved

Availability of care coordinators

Budgetary constraints

Unsure

Other, *provide details below*

……………………………………………………………………………………………………………………………………………………………………………………………………………………………………………………………………………………………………………………………………………………………………………………………………………………………………………………………………………………………………………………

**Question 8**

Select from the list your preferred option for who would coordinate the care of your patients with rare conditions?

*Tick only one:*

The patient

Carer or parent/guardian

Someone who is employed specifically as a care coordinator

Shared responsibility across the team with no single coordinator

GP

Hospital doctor

Specialist nurse

Practice or community nurse

Charity or patient support group representative

Health visitor

Genetic counsellor

Community paediatrician

Physiotherapist

Occupational therapist

Palliative Care specialist

Other, *provide details below*

**Question 9**

……………………………………………………………………………………………………………………………………………………………………………………………………………………………………………………………………………………………………………………………………………………………………………………………………………………………………………………………………………………………………………………

In this table we would like you to indicate who you would choose to coordinate each aspect of care; the patient/carer or a formal care coordinator.

| Aspect of care | Better coordinated by patient or carer  (tick box) | Better coordinated by formal care coordinator (tick box) |
| --- | --- | --- |
| Scheduling appointments |  |  |
| Liaising between health professionals |  |  |
| Liaising between health and non-health professionals (e.g. social worker, homecare) |  |  |
| Updating care plan |  |  |
| Ensuring availability of health records at appointments |  |  |
| Coordinating transitions of care |  |  |
| Liaising with patient to coordinate multi-disciplinary clinics |  |  |
| Arranging respite care |  |  |
| Other: please add |  |  |

**Comment**

Is there anything else you would like to tell us which is specific to care coordinators?

Further information specific to care coordinators

………………………………………………………………………………………………………………………………………………………………………………………………………………………………………………………………………………………………………………………………………………………………………………………………………………………………………………………………………………………………………………………………………………………..…………………………………………………………………………………………………………………………………………………………………………………………………………………………………………………………………………………………………………………………………………………………………………………………………………………………………………………………………………………………………………………………………………………………………………………………………………………………………………………………………………………………………………………………………………………………………………………………………………………………………………………………………………………………………………………………………………………………………………………………………………………………………………………………………………………………

# Section C – Care Plans

In this section we would like you to tell us about your experience of working with personalised, documented plans relating to care (we use the term **care plan**).

*NOTE: A* ***care plan*** *is a paper or electronic document which describes the services and support that is needed and should be agreed between patients, carers and professionals. The care plan may be a single document or it may be part of another record which includes non-health services such as an Educational Care and Health Plan (ECHP).*

**Question 1**

Do you use care plans as a means to document care for patients with rare conditions?

*Tick only one:*

Yes

No *Go to question 3*

Unsure *Go to question 3*

**Question 2**

Who is primarily responsible for keeping the care plan up to date?

*Tick only one:*

The patient or carer

Someone who is employed specifically as a care coordinator

Shared responsibility between professionals

No one holds responsibility

GP

Hospital doctor

Specialist nurse

Representative of patient group or charity

Practice or community nurse

Health visitor

Genetic counsellor

Community paediatrician

Physiotherapist

Other, *provide details below*

…………………………………………………………………………………………………………………………………………………………………………………………………………………………………………………………………………………………………………………………………………………………………………………………………………………………………………………………………………………………………………………………………………………………………………………………………………………………………………………………………………………………………………………………………………………………………………………………………………………………………………………………………………………………………………

**Question 3**

Select the 3 most useful items that should be included in a care plan.

*Please select 3 boxes:*

General information and a medical summary

An assessment of current health needs

An assessment of current non-health needs (e.g., social care)

Documented health goals

Transition planning for changes in care

Scheduled reviews of the care plan

Plan of care for emergency or acute episodes

Out of office hours (non-urgent) contacts

**Comment**

Is there any other information you would like to provide that is specific to care plans?

Further information specific to care plans

…………………………………………………………………………………………………………………………………………………………………………………………………………………………………………………………………………………………………………………………………………………………………………………………………………………………………………………………………………………………………………………………………………………………………………………………………………………………………………………………………………………………………………………………………………………………………………………………………………………………………………………………………………………………………………………………………………………………………………………………………………………………………………………………………………………………………………………………………………………………………………………………………………………………………………………………………………………………………………………………………………………………………………………………………………………………………………………………………………………………………………………………………………………………………………………………………………………………………………………………………………………………………………………………………………………………………………………………………………………………………………………………………………………………………………………………………………………………………………………………………………………………………………………………………………………………………………………………………………………………………………………………………………………………………………………………………………………………………………………………………………………………………………………………………………………………………………………………………………………………………………………………………………………………………………………………………………………………………………………………………………………………………………………………………………………………………………………………………………………………………………………………………………………………………………………………………………………………………………………………………………………………………………………………………………………………………………………………………………………………………………………………………………………………………………………………………………………………………………………………………………………………

# Section D – Specialist Centres

In this section we would like you to tell us about your experience of specialist centres.

*NOTE: A* ***specialist centre*** *is a centralised facility that enables patients to see a number of health professionals in one visit. Usually, they will be experts in rare and undiagnosed conditions. Non-health professionals may also see patients at the same centre.*

Question 1

Is there a specialist centre available for the majority of your patients with rare conditions?

*Tick only one:*

Yes

No *Go to Question 5*

Unsure *Go to Question 5*

Question 2

Do you work at a specialist centre for patients with rare conditions?

*Tick only one:*

Yes

No *Go to Question 5*

Question 3

Select health professionals from the list that provide care for patients with rare conditions at your specialist centre.

*Tick ALL that apply:*

Doctors who are expert in rare or undiagnosed conditions

Doctors who are expert in aspects of health (e.g. neurologist)

Specialist nurse

Psychologist

Care coordinator

Genetic counsellor

Community paediatrician

Physiotherapist

Dietician

Speech and language therapist

Behavioral therapist

Occupational therapist

Other, *provide details below:*

……………………………………………………………………………………………………………………………………………………………………………………………………………………………………………………………………………………………………………………………………………………………………………………………………………………………………………………………………………………………………………………

**Question 4**

From the list select the services provided by the specialist centre available for the majority of your patients with rare conditions.

*Tick ALL that apply:*

Appointments with an expert in rare conditions

Appointments to see different types of health professionals at the centre

Appointments to see non-health professionals (e.g., social worker)

Multiple appointments during a single visit

Appointments which are not in-person (e.g., virtual or telephone appointments)

Extended hours for appointments

Non-urgent, out-of-hours contact

Contact for acute or emergency episodes

Access to patient support groups or charities

Diagnostic and screening procedures

Support with routine admissions

Support during emergency admissions

Access to research opportunities

Other, please provide details below:

……………………………………………………………………………………………………………………………………………………………………………………………………………………………………………………………………………………………………………………………………………………………………………………………………………………………………………………………………………………………………………………

**Question 5**

We are interested in finding out why patients may not use specialist centres if they are available. Please indicate the main reasons why patients with rare conditions might choose not to use specialist centres?

*Tick ALL that apply:*

Length of time between appointments at specialist centre

Physical difficulty in travelling to specialist centre

Distance to travel to specialist centre

Cost of travel to specialist centre

Perceived lack of benefit from the specialist centre

Patient is satisfied with quality of care provided locally

Length of appointment times at specialist centre

Other, please provide details below:

……………………………………………………………………………………………………………………………………………………………………………………………………………………………………………………………………………………………………………………………………………………………………………………………………………………………………………………………………………………………………………………

Please provide reasons for your choices:

……………………………………………………………………………………………………………………………………………………………………………………………………………………………………………………………………………………………………………………………………………………………………………………………………………………………………………………………………………………………………………………

**Comment**

Is there any other information you would like to provide that is specific to specialist centres?

Further information specific to specialist centres

…………………………………………………………………………………………………………………………………………………………………………………………………………………………………………………………………………………………………………………………………………………………………………………………………………………………………………………………………………………………………………………………………………………………………………………………………………………………………………………………………………………………………………………………………………………………………………………………………………………………………………………………………………………………………………………………………………………………………………………………………………………………………………………………………………………………………………………………………………………………………………………………………………………………………………………………………………………………………………………………………………………………………………………………………………………………………………………………………………………………………………………………………………………………………………………………………………………………………………………………………………………………………………………………………………………………………………………………………………………………………………………………………………………………………………………………………………………………………………………………………………………………………………………………………………………………………………………………………………………………………………………………………………………………………………………………………………………………………………………………………………………………………………………………………………………………………………………………………………………………………………………………………………………………………………………………………………………………………………………………………………………………………………………………………………………………………………………………………………………………………………………………………………………………………………………………………………………………………………………………………………………………………………………………………………………………………………………………………………………………………………………………………………………………………………………………………………………………………………………………………………………………

# Section E - Quality of care impacts

In this section we would like you to indicate how much you agree or disagree with the statements in each table regardless of your prior knowledge or experience of these factors. RANDOMISE

| **Statement 1** | Strongly agree | Agree | Neither agree or disagree | Disagree | Strongly disagree |
| --- | --- | --- | --- | --- | --- |
| Having a care plan improves the quality of care for people with rare conditions |  |  |  |  |  |

| **Statement 2** | Strongly agree | Agree | Neither agree or disagree | Disagree | Strongly disagree |
| --- | --- | --- | --- | --- | --- |
| Having a care coordinator improves the quality of care for people with rare conditions |  |  |  |  |  |

| **Statement 3** | Strongly agree | Agree | Neither agree or disagree | Disagree | Strongly disagree |
| --- | --- | --- | --- | --- | --- |
| Attending a specialist centre improves the quality of care for people with rare conditions |  |  |  |  |  |

**Comment**

If you would like to explain any of your answers to the tables in Section E further, please do so in the box below:

……………………………………………………………………………………………………………………………………………………………………………………………………………………………………………………………………………………………………………………………………………………………………………………………………………………………………………………………………………………………………………………………………………………………………………………………………………………………………………………………………………………………………………………………………………………………………………………………………………………………………………………………………………………………………………………………………………………………………………………………………………………………………………………………………………………………………………………………………………………………………………………………………………………………………………………………………………………………………………………………………………………………………………………………………………………………………………………………

# Section F – Discrete Choice Experiment (DCE)

In the following section you will see two different scenarios. We would like you to tell us which one you would prefer for your patients, which may or may not be the one you think they would prefer.

You will be asked to do this six times, each time with a slightly different set of options, so please make sure you read each scenario carefully before ticking the option you prefer (Service A or Service B).

Each scenario will have different characteristics for six aspects of care coordination; we have listed them all below so you know what they are before you start.

| **Cost of attending all appointments over one year** | | | | | |
| --- | --- | --- | --- | --- | --- |
| Describes the cost to patients and carers of attending all health care appointments over one year (including travel costs, time off work, childcare costs, subsistence). | | | | | |
| Possible values | | | | | |
| £200 | £400 | | £1000 | | £2000 |
|  | | | | | |
| **Access to health records** | | | | | |
| Describes the way in which health records are shared by different health professionals in the same centre or across different health settings. | | | | | |
| Possible values | | | | | |
| Health records are not shared. Test results and clinic letters are sent through the post. | | | Electronic health records are immediately accessible to staff. | | |
|  | | | | | |
| **Clinical expertise** | | | | | |
| The type of medical professional who is the lead consultant and makes the majority of decisions regarding medical care. | | | | | |
| Possible values | | | | | |
| The lead consultant is a medical expert in the patient’s specific condition | | | The lead consultant is a medical expert in the area of the body primarily affected by the patient’s condition (e.g., neurologist) | | |
|  | | | | | |
| **Role of care coordinator** | | | | | |
| Describes the amount of involvement of a formal care coordinator who is a health care professional. | | | | | |
| Possible values | | | | | |
| Care is provided without the support of a care coordinator | | Care is entirely coordinated on behalf of the patient by a care coordinator | | The patient/carer decides how they wish to be supported by the care coordinator | |
|  | | | | | |
| **Access to specialist centre** | | | | | |
| A specialist centre enables patients to see a number of health professionals in one visit. Generally, they will be experts in rare and undiagnosed conditions. Non-health professionals may also see patients at the same centre*.* | | | | | |
| Possible values | | | | | |
| A specialist centre **is not** available | | | A specialist centre is available | | |
|  | | | | | |
| **Documented emergency Plan** | | | | | |
| A formal emergency plan describes the correct treatment health professionals should provide in urgent situations and contact details for a health professional who has knowledge of the specific condition. | | | | | |
| Possible values | | | | | |
| There is a documented emergency plan in place | | | No documented emergency plan exists | | |

**First an example...**You have been asked to consider the characteristics of Service A and Service B listed below and then tick the box to indicate which of the two services you would choose for your patients.

|  | Service A | Service B |
| --- | --- | --- |
| Cost of attending all appointments over one year | £200 | £1,000 |
| Access to health records | Health records are not shared. Test results and clinic letters are sent through the post. | Electronic health records are immediately accessible to staff. |
| Clinical expertise | The lead consultant is a medical expert in the patient’s specific condition | The lead consultant is a medical expert in the area of the body primarily affected by the patient’s condition |
| Role of care coordinator | Care is provided without the support of a care coordinator | Care is entirely coordinated on behalf of the patient by a care coordinator |
| Access to specialist centre | A specialist centre is not available | A specialist centre is available |
| Documented emergency plan | There is a documented emergency plan in place | No documented emergency plan exists |

If, on balance, if you would prefer Service B for the majority of your patients rather than Service A, then you would tick the box for Service B:

Service A Service B X

# Scenario 1

|  | **Service A** | **Service B** |
| --- | --- | --- |
| **Cost of attending all appointments over one year** | £200 | £1,000 |
| **Access to health records** | Health records are not shared. Test results and clinic letters are sent through the post. | Electronic health records are immediately accessible to staff. |
| **Clinical expertise** | The lead consultant is a medical expert in the patient’s specific condition | The lead consultant is a medical expert in the area of the body primarily affected by the patient’s condition |
| **Role of care coordinator** | Care is provided without the support of a care coordinator | Care is entirely coordinated on behalf of the patient by a care coordinator |
| **Access to specialist centre** | A specialist centre is **not** available | A specialist centre is available |
| **Documented emergency plan** | There is a documented emergency plan in place | No documented emergency plan exists |

Which Service would you choose? (Tick one box only)

Service A  Service B

# Scenario 2

|  | **Service A** | **Service B** |
| --- | --- | --- |
| **Cost of attending all appointments over one year** | £1,000 | £400 |
| **Access to health records** | Electronic health records are immediately accessible to staff. | Health records are not shared. Test results and clinic letters are sent through the post. |
| **Clinical expertise** | The lead consultant is a medical expert in the patient’s specific condition | The lead consultant is a medical expert in the area of the body primarily affected by the patient’s condition |
| **Role of care coordinator** | The patient/carer decides how they wish to be supported by the care coordinator | Care is provided without the support of a care coordinator |
| **Access to specialist centre** | A specialist centre is **not** available | A specialist centre is available |
| **Documented emergency plan** | No documented emergency plan exists | There is a documented emergency plan in place |

Which Service would you choose? (Tick one box only)

Service A  Service B

# Scenario 3

|  | **Service A** | **Service B** |
| --- | --- | --- |
| **Cost of attending all appointments over one year** | £1,000 | £400 |
| **Access to health records** | Electronic health records are immediately accessible to staff. | Health records are not shared. Test results and clinic letters are sent through the post. |
| **Clinical expertise** | The lead consultant is a medical expert in the area of the body primarily affected by the patient’s condition | The lead consultant is a medical expert in the patient’s specific condition |
| **Role of care coordinator** | Care is entirely coordinated on behalf of the patient by a care coordinator | The patient/carer decides how they wish to be supported by the care coordinator |
| **Access to specialist centre** | A specialist centre is **not** available | A specialist centre is available |
| **Documented emergency plan** | There is a documented emergency plan in place | No documented emergency plan exists |

Which Service would you choose? (Tick one box only)

Service A  Service B

# Scenario 4

|  | **Service A** | **Service B** |
| --- | --- | --- |
| **Cost of attending all appointments over one year** | £2,000 | £1,000 |
| **Access to health records** | Electronic health records are immediately accessible to staff. | Health records are not shared. Test results and clinic letters are sent through the post. |
| **Clinical expertise** | The lead consultant is a medical expert in the patient’s specific condition | The lead consultant is a medical expert in the area of the body primarily affected by the patient’s condition |
| **Role of care coordinator** | Care is entirely coordinated on behalf of the patient by a care coordinator | Care is provided without the support of a care coordinator |
| **Access to specialist centre** | A specialist centre is available | A specialist centre is **not** available |
| **Documented emergency plan** | No documented emergency plan exists | There is a documented emergency plan in place |

Which Service would you choose? (Tick one box only)

Service A  Service B

# Scenario 5

|  | **Service A** | **Service B** |
| --- | --- | --- |
| **Cost of attending all appointments over one year** | £200 | £2,000 |
| **Access to health records** | Electronic health records are immediately accessible to staff. | Health records are not shared. Test results and clinic letters are sent through the post. |
| **Clinical expertise** | The lead consultant is a medical expert in the patient’s specific condition | The lead consultant is a medical expert in the area of the body primarily affected by the patient’s condition |
| **Role of care coordinator** | Care is provided without the support of a care coordinator | The patient/carer decides how they wish to be supported by the care coordinator |
| **Access to specialist centre** | A specialist centre is available | A specialist centre is **not** available |
| **Documented emergency plan** | No documented emergency plan exists | There is a documented emergency plan in place |

Which Service would you choose? (Tick one box only)

Service A  Service B

|  | **Service A** | **Service B** |
| --- | --- | --- |
| **Cost of attending all appointments over one year** | £1,000 | £200 |
| **Access to health records** | Electronic health records are immediately accessible to staff. | Health records are not shared.   Test results and clinic letters are sent through the post. |
| **Clinical expertise** | The lead consultant is a medical expert in the patient’s specific condition | The lead consultant is a medical expert in the area of the body primarily affected by the patient’s condition |
| **Role of care coordinator** | Care is provided without the support of a care coordinator | The patient/carer decides how they wish to be supported by the care coordinator |
| **Access to specialist centre** | A specialist centre is **not** available | A specialist centre is available |
| **Documented emergency plan** | There is a documented emergency plan in place | No documented emergency plan exists |

# Scenario 6

Which Service would you choose? (Tick one box only)

Service A  Service B

Please tell us how easy or difficult you found this section (Section F – DCE) to complete.

Very easy

Easy

Difficult

Very difficult

If you found this section of the questionnaire difficult or very difficult to complete please tell us why in the box below.

Reason?…………………………………………………………………………………………………………………………………………………………………………………………………………………………………………………………………………………………………………………………………………………………………………………………………………………………………………………………………………………………………… ……………………………………………………………………………………………………………………………………………………………………………………………………………………………………………………………………………………………………………………………………………………………………………………………………………………………………………………………………………………………………………………………………………………………………………………………………………………………………………………………………………………………………………………………………………………………………………………………………………………

Please rank the different aspects of care coordination below in order of importance for patients with rare conditions with 1 being the attribute you consider most important for care coordination and 6 being the aspect you consider least important for care coordination.

| **ASPECTS OF COORDINATED CARE** | **DEFINITION** | RANK IN ORDER OF IMPORTANCE 1=most important  6= least important |
| --- | --- | --- |
| **Cost of attending all appointments over one year** | Describes the cost of attending all health care appointments over one year (including travel costs, time off work, childcare costs, subsistence). |  |
| **Access to health records** | Describes the way in which health records are shared by different health professionals in the same centre or across different health settings. |  |
| **Clinical expertise** | The type of medical professional who is the lead consultant and makes the majority of decisions regarding medical care. |  |
| **Role of care coordinator** | Describes the amount of involvement of a formal care coordinator who is a health care professional. |  |
| **Access to specialist centre** | A specialist centre enables patients to see a number of health professionals in one visit. Generally, they will be experts in rare and undiagnosed conditions. Non-health professionals may also see patients at the same centre. |  |
| **Documented emergency plan** | A formal emergency plan describes the correct treatment health professionals should provide in urgent situations and contact details for a health professional who has knowledge of the specific condition. |  |

# Information about you - Section G

In this section we would like to collect some information about you.

**Question 1**

Which of the following best describes your role?

**(Please tick as many as appropriate)**

Allied Health Professional

Pharmacy

Mental health / Psychological therapies

Doctor (hospital)

Doctor (GP/community)

Nursing / Midwifery

Health informatics / Healthcare science

Public Health

Management

Dentist

Ambulance services

Commissioner

Patient representative

Clinical academic

Other (please specify) ________________________________

Question 2

Which geographical region of the UK do you work in?

*Tick only one:*

| East of England  East Midlands  London  North East & Cumbria  Northern Ireland  North West of England  Scotland | South East of England  South West of England  Wales  West Midlands  Yorkshire  Prefer not to say  Other [Please specify in the box below] |
| --- | --- |

…………………………………………………………………………………………………………………………………………………………………………………………………………………………………………………………………………………………………………………………………………………………………………………………………………………………………………………………………………………………………………………

**Comment**

Is there anything else you would like to tell us about care coordination for patients with rare conditions?

Comment……………………………………………………………………………………………………………………………………………………………………………………………………………………………………………………………………………………………………………………………………………………………………………………………………………………………………………………………………………………………………………………………………………………………………………………………………………………………………………………………………………………………………………………………………………………………………………………………………………………………………………………………………………………………………………………………………………………………………………………………………………………………………………………………………………………………………………………………………………………………………………………………………………………………………………………………………………………………………………………………………………………………………………………………………………………………………………………………………………………………………………………………………………………………………………………………………………………………………………………………………………………………………………………………………………………………………………………………………………………………………………………………………………………………………………………………………………………………………………………………………………………………………………………………………………………………………………………………………………………………………………………………………………………………………………………………………………………………………………………………………………………………………………………………………………………………………………………………………………………………………………………………………………………………………………………………………………………………………………………………………………………………………………………………………………………………………………………………………………………………………………………………………………………………………………………………………………………………………………………………………………………………………………………………………………………………………………………………………………………………………………………………………………………………………………………………………………………………………………………………………………………………………………………………………………………………………………………………………………………………………………………………………………………………………………………………………………………………………………………………………………………………………………………………………………………………………………………………………………………………………………………………………………………………………………………………………………………………………………………………………………………………………………………………………………………………………………………………………………………………………………………………………………………………………………………………………………………………………………………………………………………………………………………………………………………………………………………………………………………………………………………………………………………………………………………………………………………………………………………………………………………………………………………………………………………………………………………………………………………………………………………………………………………………………………………………………………………………………………………………………

**Question 3:** Please tell us the name of the organisation which provided you with this survey**:**

**……………………………………………………………………………………………….**

Thank you so much, you made it ☺! **We hope you enjoyed taking part in this survey. Your answers will be very valuable to us**. If you have any comments, questions or suggestions about this survey or the CONCORD study in general, please write them in the box below.

Comment…………………………………………………………………………………………………………………………………………………………………………………………………………………………………………………………………………………………………………………………………………………………………………………………………………………………………………………………………………………………………………………………………………………………………………………………………………………………………………………………………………………………………………………………………………………………………………………………………………………………………………………………………………………………………………………………………………………………………………………………………………………………………………………………………………………………………………………………………………………………………………………………………………………………………………………………………………………………………………………………………………………………………………………………………………………………………………………………………………………………………………………………………………………………………………………………………………………………………………………………………………………………………………………………………………………………………………………………………………………………………………………………

If you have any questions you would like to discuss in person please contact:

Emma Hudson email:e.hudson@ucl.ac.uk

Free Telephone Number: 0800 084 2783

To find out more about the CONCORD study or to access the results of the study when they become available, please go to: https://www.geneticalliance.org.uk/our-work/healthcare-and-delivery/coordinated-care-of-rare-diseases-concord/

**Thank you again for taking part.**

# 
